# *Giardia* hinders growth by disrupting nutrient metabolism independent of inflammatory enteropathy

**DOI:** 10.1038/s41467-023-38363-2

**Published:** 2023-05-18

**Authors:** Natasa Giallourou, Jason Arnold, Elizabeth T. Rogawski McQuade, Muyiwa Awoniyi, Rose Viguna Thomas Becket, Kenneth Walsh, Jeremy Herzog, Ajay S. Gulati, Ian M. Carroll, Stephanie Montgomery, Pedro Henrique Quintela, Angela M. Faust, Steven M. Singer, Anthony A. Fodor, Tahmeed Ahmad, Mustafa Mahfuz, Esto Mduma, Thomas Walongo, Richard L. Guerrant, R. Balfour Sartor, Jonathan R. Swann, Margaret N. Kosek, Luther A. Bartelt

**Affiliations:** 1 Division of Digestive Diseases, Department of Metabolism, Digestion, and Reproduction, Faculty of Medicine, Imperial College London, London, UK; 2 Centre of Excellence in Biobanking and Biomedical Research, Molecular Medicine Research Center, University of Cyprus, Nicosia, Cyprus; 3 Center for Gastrointestinal Biology and Disease, Division of Gastroenterology and Hepatology, Department of Medicine, University of North Carolina at Chapel Hill, Chapel Hill, NC USA; 4 Department of Molecular Genetics and Microbiology, Duke Microbiome Center, Duke University School of Medicine, Durham, NC 27710 USA; 5 Department of Epidemiology, Emory University, Atlanta, GA USA; 6 Departments of Pediatrics and Pathology and Laboratory Medicine, The University of North Carolina at Chapel Hill, Chapel Hill, NC USA; 7 Institute for Infectious Diseases and Global Health and the Division of Infectious Diseases, Department of Medicine, The University of North Carolina at Chapel Hill, Chapel Hill, NC USA; 8 Department of Nutrition, Gillings School of Public Health, The University of North Carolina at Chapel Hill, Chapel Hill, NC USA; 9 Department of Pathology and Laboratory Medicine, The University of North Carolina at Chapel Hill, Chapel Hill, NC USA; 10 Institute of Biomedicine, Federal University of Ceará, Fortaleza, CE Brazil; 11 Waterborne, Inc, New Orleans, LA USA; 12 Department of Biology, Georgetown University, Washington, DC USA; 13 The University of North Carolina Charlotte, Department of Bioinformatics and Genomics, Charlotte, USA; 14 International Center for Diarrheal Disease Research, Dhaka, Bangladesh; 15 Haydom Global Health Research Centre, Haydom Lutheran Hospital, Haydom, Tanzania; 16 Division of Infectious Diseases and International Health, Department of Medicine, The University of Virginia Charlottesville, Charlottesville, VA USA; 17 School of Human Development and Health, Faculty of Medicine, University of Southampton, Southampton, UK; 18 Department of Microbiology & Immunology, University of North Carolina at Chapel Hill, Chapel Hill, NC USA

**Keywords:** Parasitic infection, Gastrointestinal diseases

## Abstract

*Giardia lamblia (Giardia)* is among the most common intestinal pathogens in children in low- and middle-income countries (LMICs). Although *Giardia* associates with early-life linear growth restriction, mechanistic explanations for *Giardia-*associated growth impairments remain elusive. Unlike other intestinal pathogens associated with constrained linear growth that cause intestinal or systemic inflammation or both, *Giardia* seldom associates with chronic inflammation in these children. Here we leverage the MAL-ED longitudinal birth cohort and a model of *Giardia* mono-association in gnotobiotic and immunodeficient mice to propose an alternative pathogenesis of this parasite. In children, *Giardia* results in linear growth deficits and gut permeability that are dose-dependent and independent of intestinal markers of inflammation. The estimates of these findings vary between children in different MAL-ED sites. In a representative site, where *Giardia* associates with growth restriction, infected children demonstrate broad amino acid deficiencies, and overproduction of specific phenolic acids, byproducts of intestinal bacterial amino acid metabolism. Gnotobiotic mice require specific nutritional and environmental conditions to recapitulate these findings, and immunodeficient mice confirm a pathway independent of chronic T/B cell inflammation. Taken together, we propose a new paradigm that *Giardia*-mediated growth faltering is contingent upon a convergence of this intestinal protozoa with nutritional and intestinal bacterial factors.

## Abstract (web summary)

*Giardia lamblia* intestinal infection is independently associated with faltering linear growth in children in low-middle income countries, yet the mechanistic pathway has not been clearly identified. Authors utilise the MAL-ED cohort, and a gnotobiotic murine model, to explain *Giardia*-induced effects on childhood growth.

## Introduction

In his diarrheal stool, Antoine van Leeuwenhoek first reported motile-flagellated ‘animalcules’ in 1681, eventually named *Giardia lamblia* in 1859^1^. After decades of pathogenic uncertainty, Theodore Nash fulfilled Koch’s postulates by showing strain-dependent diarrhea after challenge with axenized trophozoites in previously healthy adult volunteers^2^. *Giardia* is now estimated to cause > 200 million intestinal infections per year globally, and exposure to *Giardia* in children in low- and middle-income countries (LMICs) is nearly universal. Yet, the global health burden of this widely distributed parasite remains uncertain. The vast majority of pediatric infections in hyperendemic settings are without clinical symptoms^3,4^, absent intestinal pathology, and have even been demonstrated to associate with decreased risk for severe diarrhea^4,5^.

The Malnutrition and Enteric Diseases (MAL-ED) multicenter study in 8 LMICs identified asymptomatic *Giardia* detection as an independent risk factor for reduced early life linear growth in children from LMICs^3,4,6^. The hypothesized mechanism is that chronic enteric infections diminish linear growth through a process of pathogen-induced epithelial cell injury that fuels host-mediated mucosal damage and systemic inflammation, a condition termed environmental enteric dysfunction (EED). While both epithelial cell and mucosal inflammation domains and biomarkers have been defined within the EED paradigm, such as lactulose:mannitol absorption as an indicator of increased permeability of the epithelial barrier and fecal myeloperoxidase and neopterin as markers of inflammation, it remains unclear whether dysfunction in either domain alone is sufficient to impair child growth, or if both must be present. In prior MAL-ED studies, the presence of *Giardia* associated with increased small intestinal permeability indicative of epithelial cell damage, but associations with diarrhea and intestinal or systemic inflammation were absent, consistent with other studies^3,7,8^. In addition, intestinal biopsies from growth-impaired children with *Giardia* infection have shown relative decreases in lymphocyte gene expression compared with growth-impaired children without *Giardia*^9^. Thus, *Giardia* presents an example of a gastrointestinal infection causing growth failure in the absence of the EED inflammation pathway, which challenges the paradigm that EED inflammation is a final common pathway for pathogen-mediated impaired early childhood growth.

Further challenging estimates of *Giardia* effects on early child health is uncertainty surrounding the mechanistic pathway whereby *Giardia* may lead to growth impairment. In the absence of a specific biomarker of *Giardia* pathogenesis, it is difficult to reconcile seemingly conflicting associations between *Giardia* and childhood growth that vary between studies conducted in different geographic sites^3,4,6,8,10,11^. Even experimental animal models of giardiasis demonstrate phenotypes ranging from diarrhea, chronic enteropathy, and no apparent pathology that are not entirely explainable by defined differences between parasite or mouse strains^5^. These findings have led the field to hypothesize that factors in addition to parasite and host genetics determine consequences of *Giardia* infection^5^. For example, we previously published that experimental challenge with human *Giardia* assemblage B strain H3 cysts can impair weanling growth in conventionally raised protein-deprived mice^12^, but continuous antibiotics that deplete intestinal microbiota can prevent this growth restriction^13^. These data suggest that environmental factors, like diet and other microbes in the small intestine contribute to *Giardia* pathogenesis^13–19^.

In this work, we perform translational investigations using the MAL-ED cohort and a novel gnotobiotic murine model to identify mechanistic pathways that may explain *Giardia*-induced effects on early childhood growth. We demonstrate the consequences of environmental influences on experimental *Giardia*-mono-association infection in a gnotobiotic mouse model correspond with reductions in free amino acids but are independent from intestinal lymphocytic inflammation typical of EED. Rather, we identify signatures in the urinary metabolome of young children which suggest that host growth restriction during *Giardia* infection is mediated by dysregulated amino acid metabolism suggestive of diminished host amino acid absorption with microbial conversion to potentially toxic byproducts.

## Results

### Quantitative *Giardia* burden estimates on early childhood linear growth and intestinal permeability

The application of standardized quantitative molecular diagnostics in multi-site studies has improved confidence of estimates of pathogen-attributable diarrhea burdens amidst frequent co-pathogen exposures in children in LMICs^4,20,21^. In the MAL-ED multi-site investigation, shared fecal collection, processing, and EED biomarker assay protocols coupled with frequent site visits for protocol adherence also minimized methodological differences between sites allowing for both combined and site-specific analysis^22^. Further, in a sub-group of MAL-ED participants from Peru and Bangladesh additional profiling of serum amino acids (15 months-of-age) and serial urine collections for metabolomics were performed^20,23^.

**Fig. 1:**
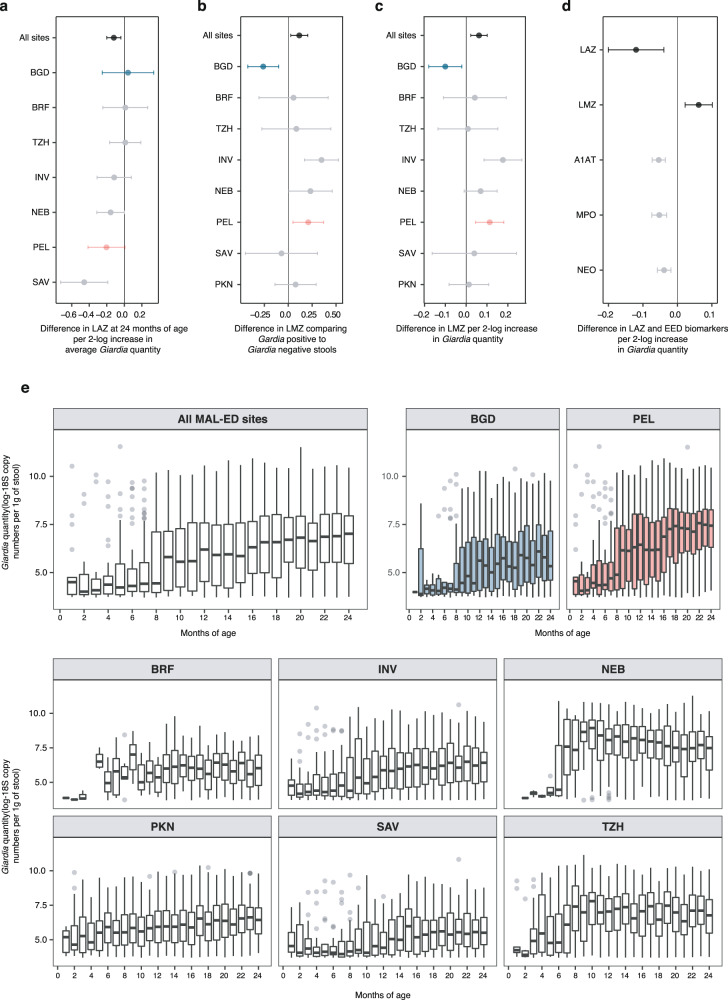
Associations between quantitative *Giardia* burden and growth impairment and small intestinal permeability. **a** Association between *Giardia* and length-for-age z-score (LAZ) at 24 months across all MAL-ED sites. Estimates presented are adjusted for enrolment LAZ, sex, socioeconomic status, maternal height, exclusive breastfeeding duration in the first six months of life and additionally adjusted for *Campylobacter, Shigella, enteroaggregative Escherichia coli and Enterocytozoon bieneusi*. Association between *Giardia* detection (**b**) or *Giardia* quantity (**c**) and lactulose:mannitol z-score (LMZ). Estimates are adjusted for age, sex, socioeconomic status and exclusive breastfeeding duration in the first six months of life and additionally adjusted for *Enterocytozoon bieneusi, Cryptosporidium*, atypical enteropathogenic *E coli*, Norovirus, Astrovirus and Sapovirus. **d** Associations between *Giardia* and fecal environmental enteric dysfunction (EED) biomarkers, alpha-1-anti-trypsin (A1AT), myeloperoxidase (MPO), and neopterin (NEO). Estimates have been adjusted for age, sex, socioeconomic status, exclusive breast feeding in the first six months and stool consistency. Estimates are additionally adjusted for co-pathogens that associate with each respective biomarker at a *p* < 0.05 level (see methods). LAZ and LMZ estimates presented here are identical to those of figures a and **c**. **a**–**d** Show mean difference point estimates and 95% confidence intervals: **a** All sites (*N* = 1469 individual participants), Bangladesh (BGD; *N* = 210), Peru (PEL; *N* = 194), Brazil (BRF; *N* = 165), India (INV; *N* = 227), Nepal (NEB; *N* = 227), Pakistan (PKN; *N* = 246), South Africa (SAV; *N* = 237), Tanzania (TZH; *N* = 209); **b**, **c** All sites (*N* = 3160 biologically independent measurements); BGD (*N* = 525); PEL (*N* = 337); BRF (*N* = 229); INV (*N* = 529); NEB (*N* = 601); PKN (*N* = 393); SAV (*N* = 345); TZH (*N* = 201) (**d**) LAZ (*N* = 1469 individual participants); LMZ (*N* = 3160 biologically independent measurements); A1AT, MPO, and NEO (*N* = 19,009 biologically independent measurements for each target). **e** *Giardia* quantitative burden in non-diarrheal stools obtained from 1715 children across all MAL-ED sites (left panel) from birth to 24 months of age and each specific site: Bangladesh (*N* = 210), Peru (*N* = 194), Brazil (*N* = 165), India (*N* = 227), Nepal (*N* = 227), Pakistan (*N* = 246), South Africa (*N* = 237), Tanzania (*N* = 209) (median ± IQR). (LAZ data for Pakistan were excluded due to bias identified in a subset of the measurements^24^). All data were analyzed from biologically independent samples. Source data are provided as a Source Data file.

We used these data to examine the extent to which quantitative *Giardia* burden influences the estimates of associations between *Giardia* and linear growth, intestinal permeability, and intestinal inflammatory biomarkers of EED. After adjusting for enrollment length-for-age z-score (LAZ), sex, socioeconomic status, exclusive breastfeeding duration in the first 6 months of life, maternal height, and additional adjustment for *Campylobacter, Shigella, enteroaggregative Escherichia coli and Enterocytozoon bieneusi*, overall, across all sites there was a −0.12 (CI: −0.20, −0.04) reduction in attained LAZ at 24 months-of-life per 2 log increase in average *Giardia* quantity in non-diarrheal stools collected over the duration of the study (Fig. 1a, Supplementary Fig. 1). Similarly, increased small intestinal permeability measured as lactulose:mannitol z-scores (LMZ) associated with concurrent *Giardia* detection (Fig. 1b, Supplementary Fig. 1) and per 2 log increase in *Giardia* quantity (Fig. 1c, Supplementary Fig. 1) adjusted for age, stool consistency, sex, socioeconomic status, exclusive breastfeeding duration in the first 6 months of life, and co-pathogens associated with LMZ (*Enterocytozoon bieneusi*, *Cryptosporidium*, atypical enteropathogenic *Escherichia coli* (EPEC), Norovirus, Astrovirus, and Sapovirus). Across combined sites, LMZ was the only biomarker that positively associated with quantitative *Giardia* burden, whereas the fecal EED biomarkers inversely associated with *Giardia* quantitative burden (Fig. 1d, Supplementary Fig. 1).

Across combined MAL-ED sites, *Giardia* burden increased with age (Fig. 1e), even though the strength of *Giardia* associations with LAZ and LMZ showed significant inter-site variability (Fig. 1a–c). For example, in Peru, where 85.6% of children had a *Giardia* infection by 12 months of age, strong associations between *Giardia* and reduced LAZ and increased LMZ were found. In contrast, in Bangladesh at 15 months of age, prevalence (Bangladesh: 24%, Peru: 51%) and quantity of *Giardia* detection was significantly lower than in Peru (Mann-Whitney U-test, *P* < 0.0001), and neither LAZ nor LMZ was associated with *Giardia* detection (Fig. 1a–c). At other sites Nepal, India, and South Africa resembled effects seen in Peru, whereas Tanzania, Brazil, and Pakistan resembled Bangladesh. Enrollment characteristics^4^ (including sex, improved sanitation, improved drinking water, maternal height, maternal age, monthly income, months of exclusive breast feeding, enrolment LAZ, and urban versus rural setting) did not consistently differ in respect to sites with or without *Giardia-*specific LAZ and LMZ associations. In addition, assemblage-level *Giardia* genotype frequencies have previously been reported to be similar between all MAL-ED sites^3^.

### Dietary and environmental factors mediate vulnerability to growth restriction and small intestinal permeability outcomes during *Giardia* infection

**Fig. 2:**
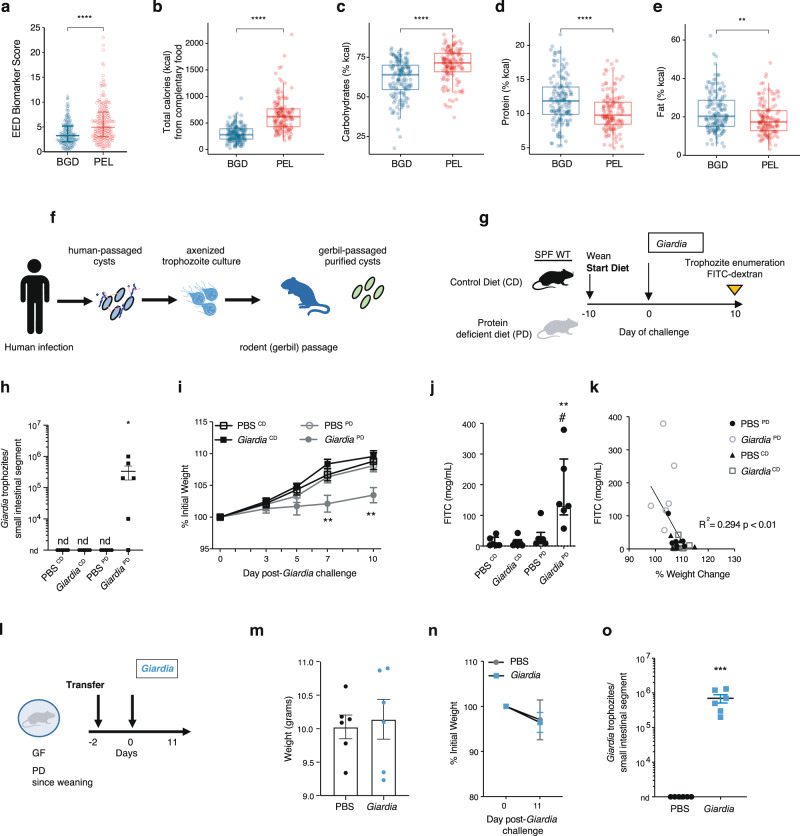
Diet and environmental factors enhance vulnerability to *Giardia* mediated growth faltering and gut dysfunction. **a** Fecal EED Biomarker score in independent samples from children from Bangladesh (BGD; *N* = 183) and Peru (PEL; *N* = 145) at 15 months of age (median ± IQR, ^****^P < 0.0001, two-sided Mann-Whitney U-test). **b** Amount of total calories ingested from complimentary foods at 15 months of age. Proportion (%) of calories derived from (**c**), carbohydrates (**d**), protein and (**e**), fat at 15 months of age. For (**b**–**e**), data represent biological independent samples (median ± IQR), **b** ****P < 2.22e-16, **c** ****P = 1.1e-10, **d** ****P = 1.2e-05, **e** **P = 0.0023, two-sided Mann-Whitney U-tests comparing Bangladesh (*N* = 158) and Peru (*N* = 150). **f** Schematic of commercial *G. lamblia* (*Giardia*) H3 cysts preparation. **g** Timeline for specific pathogen free (SPF) mice experiment (**h**–**k**). **h** Enumeration of *Giardia* trophozoites from the upper small intestine by light microscopy (mean ± SEM; nd=none detected; *P = 0.046 for PD-*Giardia* vs all other groups, One-way ANOVA, Holm-Sidak’s multiple comparisons test). **i** Growth as a percentage of initial weight on the day of PBS or *Giardia* cyst (10^5^) oral gavage challenge (day 0) (mean ± SEM; **P < 0.01 for PD *Giardia* vs all other groups, Two-way ANOVA, repeated measures, Bonferroni multiple comparisons test. See Source Data File for exact *P*-values). **j** Serum FITC detection (median ± IQR; **P = 0.005 PD-*Giardia* vs CD-*Giardia* and ^#^*P* = 0.011 for PD-*Giardia* vs CD indicated groups, Kruskal-Wallis, Dunn’s multiple comparisons test; **P = 0.004 PD *Giardia* vs PD, two-sided Mann-Whitney U-test). **k** Simple linear regression of serum FITC concentration and % initial weight on day 10 (*R*^2^ = 0.294, P = 0.0062). **l** Timeline for germ-free (GF) mice experiment (8–13 weeks-old) (**m**–**o**). GF mice were weaned onto the PD diet and then acclimated to the SPF environment for 2 days prior to challenge. **m** Weight of mice on the day of *Giardia* 10^5^ cyst or PBS challenge (mean ± SEM). **n** Growth as percent weight change from the day of challenge (mean ± SEM). **o** Enumeration of *Giardia* trophozoites from the upper small intestine by light microscopy (mean ± SEM; ***P = 0.004, *Giardia* vs PBS, two-sided Unpaired *t*-test). For **h**–**k**, and **n**, **o**, data represent 6 biologically independent animals or samples per group over each independent experiment. Source data are provided as a Source Data file.

Given that we had additional serum amino acids at 15-months-of-age and urinary metabolomics data from Peru and Bangladesh, we focused further comparisons between these two sites that contrasted with respect to *Giardia* associations with LAZ and LMZ. Starting with dietary feeding comparisons, by 15 months-of-age, all children in both sites experienced introduction of complementary foods, however, dietary feeding practices revealed earlier introduction of non-breast milk exposures in Peru (median day of exclusive breastfeeding interruption: 20 (CI: 14.4, 25.6) Peru and 104 (CI: 97.4, 112) BGD, *P* < 0.0001) as well as a shorter duration of breastfeeding (median day of breastfeeding cessation: 536 (CI: 516, 556) Peru and 735 (CI: 714, 756 Bangladesh, *P* < 0.0001) in Bangladesh) (Supplementary Fig. 2a). At 15 months-of-age, composite fecal EED-scores (fecal neopterin, myeloperoxidase, alpha-1-anti-trypsin) were greater in children in Peru compared with children in Bangladesh (Fig. 2a), but these scores did not differ by *Giardia* status at either site (Supplementary Fig. 2b). The increased *Giardia* quantitative burden among *Giardia*-positive children at 15 months-of-age (Fig. 1e) in Peru was specific to *Giardia*, as quantitative burdens of other detectable pathogens were similar between the two sites at this timepoint (Supplementary Table 1). Children recorded a greater number of total kilocalories (kcal) from non-breast milk sources in Peru compared with children in Bangladesh (Fig. 2b). Carbohydrates accounted for the greater proportion of kcal intake in Peru (Fig. 2c), whereas the proportion of kcal from protein and to a lesser extent fat were greater in Bangladesh (Fig. 2d, e). Thus, at the population-level, greater estimates of *Giardia*-mediated disease converged with indicators of greater EED biomarkers and different complementary feeding practices.

MAL-ED investigators have previously published that lower dietary protein intake was associated with linear growth restriction and may worsen pathogen-mediated effects^24^. Similarly, in experimental models, we and others have shown that susceptibility to EED and *Giardia* infection are increased when mice are fed isocaloric, but protein-poor diets^12,13,25,26^. To test the biological plausibility that nutritional and/or environmental factors influence susceptibility to *Giardia*-mediated growth divergence together with intestinal permeability defects, we adapted our experimental *Giardia* infection model in conventionally housed (Conv) mice^12,13,25^ to a cubicle-contained individually ventilated caging (IVC) system in a specific pathogen free (SPF) gnotobiotic barrier facility. Weight-matched post-weanling three-week-old SPF-reared mice were challenged with 10^5^ commercially purified *G. lamblia* cysts (assemblage B, H3) (Fig. 2f) 10 days after acclimation to either a low protein diet (2%, PD) or isocaloric normal protein (20%) control diet (CD) (Fig. 2g, Supplementary Fig. 3a). As in Conv mice^12,13^, extended infection (Fig. 2h) and weight divergence (Fig. 2i) in IVC-housed mice required the PD diet. The additional weight restriction seen in PD diet-fed *Giardia*-infected mice (*p* < 0.05 day 7 and *p* < 0.01 day 10) (Fig. 2i) was coupled with a > 5-fold increase in intestinal permeability compared with PD diet-fed uninfected controls (Fig. 2j). Compared with CD diet-fed mice, protein deficiency and *Giardia* converged to result in a > 13-fold increase in intestinal permeability (Fig. 2j). Among all mice, intestinal permeability associated with percent growth (R^2^ = 0.294, *p* < 0.01), albeit with a wide range of within-group variability in the *Giardia-*infected PD diet-fed mice (Fig. 2k).

Nutritional status (like dietary protein or fat intake) and antibiotics that disrupt intestinal microbial communities can alter experimental *Giardia* challenge outcomes^12–14,27^ and conversely, experimental *Giardia* challenge may alter intestinal microbial community composition. To test whether intestinal microbiota mediated experimental *Giardia* infection outcomes during protein deprivation, we adapted our model in germ-free (GF) mice. GF mice were weaned onto the protein deficient diet and then transferred from isolator-housing (Iso) to IVC conditions 2 days prior to 10^5^ *Giardia* cyst challenge (Fig. 2l) in weight-matched groups (Fig. 2m). In contrast to SPF-raised mice, the PD-diet fed GF mice did not develop weight divergence after *Giardia-*challenge (Fig. 2n) despite a similar *Giardia* infection burden to SPF mice (Fig. 2o) and similar fecal weights between all groups (Supplementary Fig. 3b). In a separate experiment, we challenged control and PD diet-fed GF mice with 10^4^ cysts. Regardless of diet, these mice developed persistent infection through three weeks and at similar trophozoite burdens (Supplementary Fig. 3c), indicating that absent intestinal microbiota, the PD diet did not directly alter host susceptibility to *Giardia* colonization in this timeframe. Rather, these data supported that environment-dependent interactions with resident intestinal bacteria may mediate the increased susceptibility to more prolonged *Giardia* infection and differential weight outcomes in *Giardia*-challenged protein-deprived SPF mice.

### *Giardia* synergizes with protein deficiency to impair host growth independent from EED-like inflammation

**Fig. 3:**
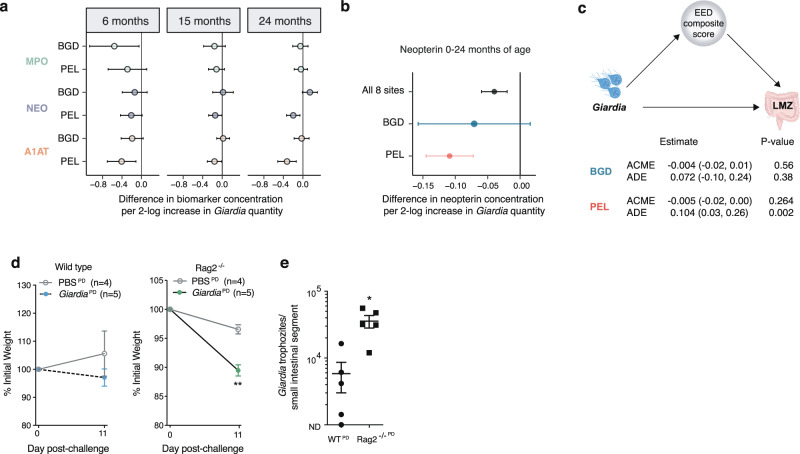
*Giardia* does not associate with inflammatory markers of EED in children, and weight loss in experimental *Giardia* challenge is greater in mice lacking T/B cells. **a** Association between EED fecal individual biomarker concentration and concurrent *Giardia* quantity at 6 (BGD *N* = 120; PEL *N* = 87), 15 (BGD *N* = 158; PEL *N* = 98), and 24 (BGD *N* = 172; PEL *N* = 139) for each target. **b** Association between neopterin concentration and *Giardia* quantity 0–24 months of life, across all MAL-ED sites (*N* = 19,009), BGD (*N* = 2574) and PEL (*N* = 2114). For (**a**, **b**) figures show mean difference point estimates and 95% confidence intervals. All data were analyzed from biologically independent samples. **c** Causal mediation analysis in biologically independent samples revealed that intestinal permeability (expressed as LMZ) as a result of *Giardia* infection in not significantly mediated by the EED composite biomarker score that is calculated based on the levels of A1AT, MPO and neopterin which are markers of intestinal inflammation and barrier disruption. Covariates included in the modelling process included sex, age and socioeconomic status. Only children with matching A1AT, MPO, neopterin, LMZ and *Giardia* detection data were included in the analysis. To facilitate comparisons between Peru and Bangladesh only matching data from measurements at 3, 6, 9, and 15 months were included in the models since no LMZ measurements were available for Bangladesh at 24 months. **d**, **e** 10^3^ commercial *Giardia* cyst challenge in biologically independent wild-type (WT) or *Rag2*^*−/−*^ germ-free (GF) mice (10–13 week-old) fed the protein deficient diet (PD) (experimental timeline details in Supplementary Fig. 5). **d** Weight as percent change after PBS (WT (N = 4), *Rag2*^*−/−*^ (*N* = 5)) or *Giardia* challenge (WT (*N* = 4), *Rag 2*^*−/−*^ (*N* = 5)) (mean ± SEM; **P < 0.0001 *Rag2*^*−/−*^ PBS vs *Giardia*, Two-way ANOVA repeated measures, Bonferroni multiple comparisons test). **e** Enumeration of *Giardia* trophozoites from the upper small intestine in biologically independent mice (mean ± SEM; nd = none detected; *P = 0.006, two-sided Unpaired t-test, N = 5 per group). Source data are provided as a Source Data file.

Although associated with chronic intestinal inflammation in sporadic outbreaks in non-endemic settings^28^, endemic pediatric *Giardia* detection is consistently not associated with EED-biomarkers, including neopterin (NEO), the marker most closely related to chronic T cell pro-inflammatory pathways observed in EED^7,29,30^. Similarly, whereas composite EED scores were increased in the presence of higher quantities of enteroaggregative *E. coli, Shigella* and *Campylobacter* detection in MAL-ED sites, *Giardia* was not (Supplementary Fig. 2c). Moreover, *Giardia* quantitative burden tended to inversely associate with concentrations of each of the component fecal EED biomarkers in multiple age-stratified analyses (6, 15, and 24 months of age): alpha-1 anti-trypsin (A1AT, a marker of protein leak into the intestine), myeloperoxidase (MPO, a marker of myeloid inflammation), and NEO, particularly in Peru (Fig. 3a). For neopterin, this pattern was consistent across all sites and timepoints in the MAL-ED cohort: the quantitative burden of *Giardia* detection by qPCR inversely associated with concurrent NEO concentration (−0.04; −0.06,−0.02 log [nmol/L]) (Fig. 3b, Supplementary Fig. 1d). This association was stronger in Peru than Bangladesh (Fig. 3b). Mediation analyses failed to identify fecal intestinal EED biomarkers that mediated the association between *Giardia* and LMZ, including either composite EED biomarkers (Fig. 3c) or each of the individual component markers (Supplementary Fig. 4). Thus, although estimates of *Giardia* effects on LAZ and LMZ in MAL-ED were stronger in a population with overall greater composite EED scores, and the effect of *Giardia* on LAZ was greater in children with higher composite EED scores (Supplementary Fig. 2d), quantitative *Giardia* burden analyses strongly uncoupled greater concentration of EED inflammatory biomarkers as a result of greater quantities of *Giardia*.

**Fig. 4:**
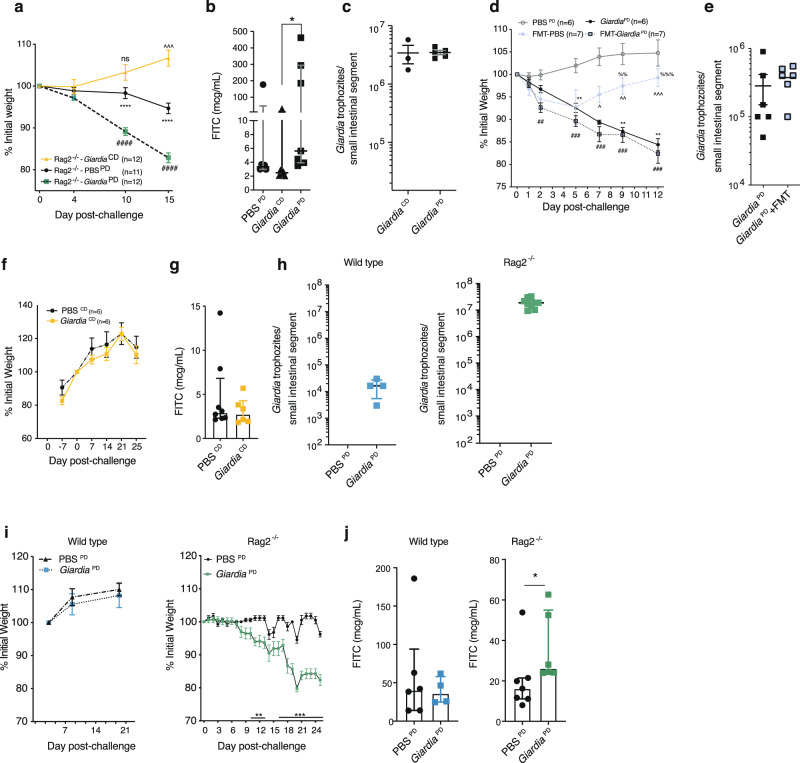
Protein deficiency is required for *Giardia-*mediated weight loss and increased intestinal permeability in immunodeficient mice. **a**–**c** Cubicle-housed GF-*Rag 2*^*−/−*^ mice fed either PD or control diet (CD) and challenged with either PBS or 10^5^-commercial *Giardia* cysts as indicated (**a**). Weights as percent change after challenge, two independent experiments: PD-PBS (*N* = 11, cohort-1 (*N* = 5), cohort-2 (*N* = 6); PD-*Giardia* (*N* = 12 cohort-1 (*N* = 5), cohort-2 (*N* = 7)), CD-*Giardia* (*N* = 12, cohort-1 (*N* = 3), cohort-2 (*N* = 9)) (mean ± SEM; ^^^^^*P* = 0.001 CD-*Giardia* vs PD-PBS; ****P < 0.0001 PD-*Giardia* vs PD-PBS, ^####^*P* < 0.0001 CD-*Giardia* vs PD-*Giardia*. Exact *P*-values provided in source data file. Details for each independent experiment in Supplementary Fig. 5). **b** Serum FITC concentration on day 15 (cohort-1) (median ± IQR; *P = 0.0067 *Giardia*^CD^ (*N* = 6, 3 mice excluded due to insufficient sample) versus *Giardia*^PD^ (*N* = 7), Kruskal-Wallis, Dunn’s multiple comparisons test; *P* = 0.035 for PBS^PD^ vs *Giardia*^PD^ two-sided Mann-Whitney U-test). **c** Trophozoites in the upper small intestine on day 15 (cohort-2) (CD (*N* = 3), PD (*N* = 5)) (mean ± SEM). **d** Weights as percent change in PD-fed mice after challenge with *Giardia* (*N* = 6), fecal microbiota transfer (FMT) from PD-fed SPF mice (*N* = 7), both (*N* = 7), or PBS (*N* = 6) (mean ± SEM; **P = 0.003 *Giardia* vs PBS; ^##^*P* = 0.004, ^###^*P* = 0.0007 *Giardia-*FMT vs PBS; ^^^*P* = 0.02, ^^^^*P* = 0.005, ^^^^^*P* = 0.0005 *Giardia-*FMT vs FMT; ^%%^*P* = 0.006, ^%%%^*P* = 0.0006 *Giardia* vs FMT). **e** Trophozoites in the upper small intestine on day 12 (mean ± SEM, *Giardia* (*N* = 6), *Giardia*-FMT (*N* = 6, one mouse excluded due to a missing sample). **f**–**j** 10^3^ axenic *Giardia* cyst challenge in isolator-housed GF-*Rag 2*^*−/−*^ mice or GF-WT mice fed either CD or PD as indicated. **f**, **g** CD-fed mice. **f** Weights as percent change beginning 7 days prior to challenge (mean ± SEM, PBS (*N* = 5), *Giardia* (*N* = 6)). **g** Serum FITC concentration on day 25 (median ± IQR, PBS (*N* = 8), *Giardia* (*N* = 6)). **h**–**j** (*left*) WT mice fed PD diet since weaning (PBS (*N* = 6), *Giardia* (*N* = 4; excluded is one mouse with immediate gavage trauma) through day 21 post-challenge and (*right*) *Rag 2*^*−/−*^ mice fed PD diet for 7 days prior to challenge and through day 25 post-challenge (PBS(*N* = 8), *Giardia* (*N* = 9)). **h** Trophozoites in the small intestine. **i** Weights as percent change after challenge (mean ± SEM, **P = 0.003 day 11, 0.004 day 12, 0.001 day 13, and ***P < 0.0001 day 16, 18–25 (*P* = 0.0002 day 17) *Rag 2*^*−/−*^ PBS versus *Rag 2*^*−/−*^ *Giardia*). **j** Serum FITC concentration (median ± IQR, excluded are one PBS-challenged for *N* = 7 and three *Giardia*-challenged mice for *N* = 6 and due to insufficient sample, *P = 0.022, two-sided Mann-Whitney U-test). **a**, **d**, **i** Two-way ANOVA, repeated measures, Bonferonni multiple comparisons test. Data are from biologically independent mice/samples from each independent experiment. Source data are provided as a Source Data file.

Experimental models in well-nourished mice demonstrate that *Giardia*-induced changes in epithelial cell morphometry, subcellular structure, and digestive function requires the presence of T cells^31–34^. However, histopathology in PD and control diet-fed IVC-housed mice supported prior findings in Conv-housed mice^13^ that *Giardia*-mediated enteropathy was unlikely to occur through EED-like inflammation: villus and crypt architecture and inflammatory infiltrates between *Giardia-*infected PD diet-fed SPF mice were indistinguishable from uninfected controls at 11 days post-challenge (Supplementary Fig. 3e). To reconcile this conundrum with our findings we used *Rag2*^*−/−*^ mice that lack T/B cell mediated inflammation to test whether *Giardia*-mediated enteropathy during protein deprivation required chronic lymphocytic activity. Whereas wild-type GF protein deprived mice showed little growth divergence, *Rag2*^*−/−*^ protein deprived mice developed significant weight loss and greater *Giardia* burden (Fig. 3d, e). In GF *Rag2*^*−/−*^ mice, the PD diet was necessary for *Giardia*-induced weight loss (Fig. 4a) and increased intestinal permeability (Fig. 4b). The interaction between *Giardia* and protein deprivation in GF *Rag2*^*−/−*^ mice drove disease severity without increasing quantitative *Giardia* burden compared with control-diet fed GF *Rag2*^*−/−*^ mice (Fig. 4c). To test whether PD diet-fed *Rag2*^*−/−*^ mice were highly vulnerable to weight loss following any microbial challenge, we conventionalized them with resident murine microbiota. Oral gavage with commensal microbiota led to transient weight loss, but unlike challenge with *Giardia* cysts, the weight loss was not sustained (Fig. 4d, e) (Supplementary Fig. 5).

While *Rag2*^*−/−*^ mice confirmed that T/B cell inflammation typical of EED was not required for commercial *Giardia* cyst-mediated weight loss, relying on commercially derived *Giardia* cysts limited our ability to definitively exclude the role of trace bacterial and fungal contaminants present in the gerbil-passaged preparation on outcomes in GF mice. To completely remove non*-Giardia* microbial influences in a natural cyst-initiated infection model, we developed a novel *Giardia* mono-association for propagation of axenic cysts in GF mice (Supplementary Fig. 3f) Using high-resolution density gradients, high-concentration detergents, and supplemental antimicrobials (ampicillin) and antifungals (micafungin) we selectively eliminated residual culturable contaminants that were transmissible into GF mice without harming cyst integrity (Supplementary Fig. 6). We then identified an ID_50_ of 10^2^ ‘ultra-pure’ cysts in GF *Rag 2*^*−/−*^ mice (Supplementary Fig. 6), and validated *Giardia* mono-association using conventional gnotobiotic methods for bacteriology: Gram’s stain, culture, and 16 S PCR (Supplementary Fig. 7)^35^. In addition, we confirmed absence of other eukaryotes using culture-based methods and microscopy for fungi and other protozoa (Supplementary Fig. 7). In mono-association, *Giardia* trophozoites were distributed with greater density in the upper small intestine compared with the lower small intestine, as expected (Supplementary Fig. 3g). Parasite concentrations were 2 logs greater in abundance in the mucosal-associated fraction than rinsed luminal contents, and trophozoites were seen along the entirety of the villus-crypt axis (Supplementary Fig. 3h). Cysts were identified beginning 7 days after propagation and reached a consistent concentration of ~10^4^ cysts per fecal pellet (Supplementary Fig. 3i, j) that persisted indefinitely ( > 142 days). We observed no cross-contamination of separately caged GF *Rag2*^*−/−*^ mice simultaneously housed within the same isolator (Supplementary Fig. 7). In PD-diet fed SPF mice, 10^4^ axenic cysts led to similar intestinal trophozoite burdens 10 days after challenge (Supplementary Fig. 3) compared with 10^5^ commercial cyst challenge (Fig. 2h).

Using the gnotobiotic-derived axenic cysts, we next performed a series of experiments in *Giardia* mono-associated mice and compared growth and intestinal permeability outcomes with contemporaneous uninfected controls within the same GF isolator. When fed a normal diet, Iso-housed *Giardia* mono-associated *Rag2*^*−/−*^ mice showed no apparent signs of distress, grew identical to uninfected GF controls, and showed no alterations in intestinal permeability (Fig. 3f, g). Axenic cyst challenge in PD diet-fed GF mice re-capitulated weight loss and increased permeability only in more heavily infected *Rag2*^*−/−*^ mice compared with WT mice (Fig. 4h–j). Mouse sex was not a determinant of phenotypes in these *Giardia* mono-association experiments. Histopathology of *Giardia* mono-associated PD-fed *Rag2*^*−/−*^ mice showed no significant changes in villus or crypt morphometry to account for the weight loss, and fecal weights at the termination of the experiment were identical between the two groups (Supplementary Fig. 8). Thus, this gnotobiotic model demonstrated biological plausibility that *Giardia*, a non-invasive protozoan confined to the small intestine, mediated pathology through a mechanistic pathway different from prototypic EED-like inflammation.

### *Giardia* promotes amino acid deficiency and alters microbial-host co-metabolism of aromatic amino acids in vulnerable populations

**Fig. 5:**
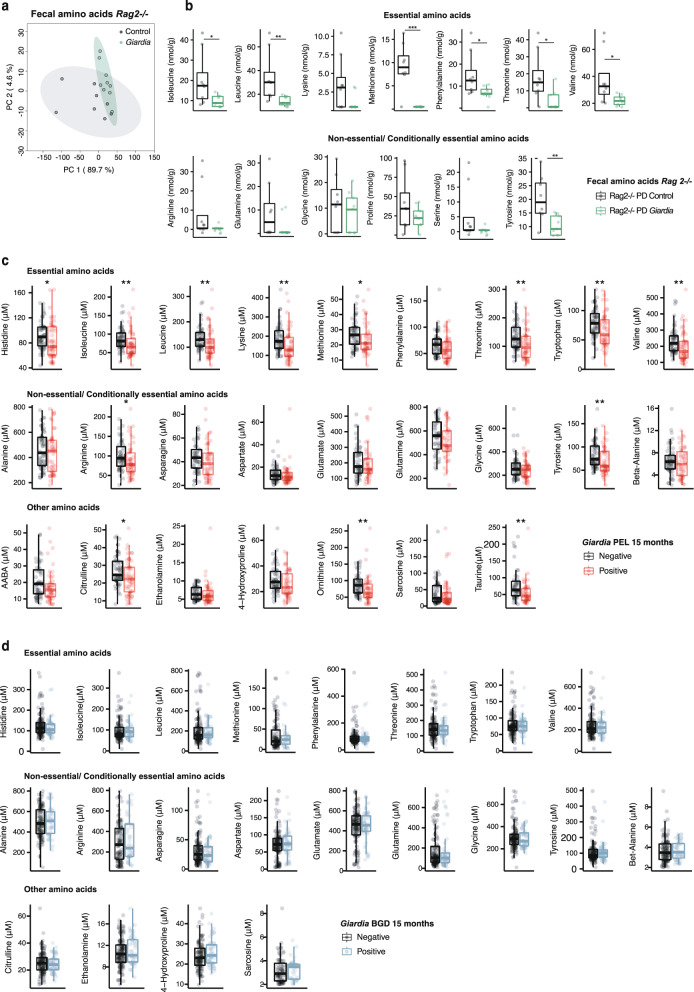
*Giardia* diminishes free amino acids in protein deprived mice, and early childhood *Giardia* infection differentially alters serum free amino acid profiles in geographically distinct populations. **a** PCA score plot comparing fecal amino acids profiles between infected and uninfected *Rag2*^*−/−*^ mice fed a PD diet. **b** Concentrations of fecal amino acids in biologically independent *Rag2*^*−/−*^ mice fed a PD diet 25 days after *Giardia* (*N* = 9) or PBS (*N* = 8)-challenge (median ± IQR; *P < 0.05 (*P* = 0.021 for isoleucine and phenylalanine, *P* = 0.011 for threonine, *P* = 0.015 for valine), **P < 0.01 (*P* = 0.0037 for leucine, *P* = 0.0079 for tyrosine), ***P = 0.0008 for methionine for PBS vs *Giardia*, two-sided Mann-Whitney U-test). **c** Concentrations of circulating amino acids measured in biologically independent plasma samples from children from Peru with concurrent *Giardia* detection at 15 months (median ± IQR, *N* = 49 with *Giardia* detection, *N* = 53 without *Giardia* detection). **d** Concentrations of circulating amino acids measured in biologically independent plasma samples from children from Bangladesh with concurrent *Giardia* detection at 15 months (median ± IQR, *N* = 46 with *Giardia* detection, *N* = 117 without *Giardia* detection). In Bangladesh, lysine, alpha-aminobutyric acid, ornithine and taurine were below the set limits of detection. For (**b**–**d**), statistical significance was determined using Mann-Whitney U-tests. For (**b**) *P < 0.05 (isoleucine *P* = 0.021, phenylalanine *P* = 0.021, threonine *P* = 0.011, valine *P* = 0.015; **P < 0.01 (leucine *P* = 0.0037, tyrosine *P* = 0.0079); ***P = 0.00084 for methionine). For (**c**), Histidine *P* = 0.027, Isoleucine *P* = 0.0037, Leucine *P* = 0.0056, Lysine *P* = 0.0022, Methionine *P* = 0.35, Threonine *P* = 0.002, Tryptophan P = 0.0057, Valine *P* = 0.0028, Arginine *P* = 0.029, Tyrosine *P* = 0.025, Citrulline *P* = 0.034, Ornithine *P* = 0.002, Taurine *P* = 0.0038. Source data are provided in the Source Data file.

Because neither MAL-ED cohorts nor the mouse model indicated that *Giardia-*mediated growth restriction occurred as a result of increased intestinal inflammation, we alternatively hypothesized that *Giardia-*mediated growth restriction resulted from parasite-mediated metabolic disruptions that could result from nutrient competition or interfere with homeostasis of nutrient digestion and absorption during limited nutrient availability. We therefore analyzed amino acid profiles in feces and serum of mice using a targeted liquid chromatography with amino acids standards. *Giardia* significantly altered the free amino acid (AA) profiles of PD diet-fed mono-associated *Rag2*^*−/−*^ mice (Fig. 5a) resulting in depletion of multiple AAs in the intestinal compartment (Fig. 5b). Serum AA concentrations were overall very low in these mice, but *Giardia* similarly tended to further decrease concentrations of several AA, significantly for threonine, indicating that increased absorption of AA could not explain reduced free AA in the intestinal compartment^36^ (Supplementary Fig. 9).

To determine if *Giardia* altered AA metabolism in humans, we analyzed circulating AAs using LC-MS available in the subset of 15-month-old children in Peru and Bangladesh. Plasma AA profiles revealed significant inverse associations between citrulline and tryptophan and LMZ (R^2^ = −0.17 and *P* = 0.0032, R^2^ = −0.14 and *P* = 0.019 respectively) irrespective of study site and infection status. Further, levels of several other free serum AA were diminished in children with concurrent *Giardia* detection in Peru but not in Bangladesh (Fig. 5c, d). This included reductions in essential AAs histidine, lysine, methionine, threonine, and tryptophan, and the branched-chain AAs, isoleucine, leucine, and valine. Among the non-essential/conditional amino acids, the presence of *Giardia* associated with a decrease in a more restricted subset: arginine and other AAs involved in arginine metabolism (citrulline and ornithine), tyrosine, and taurine (Fig. 5c, d). Thus, consistent with inter-site variability in associations between *Giardia* and poor growth attainment, these observations were site-specific and only significant in the Peru cohort.

**Fig. 6:**
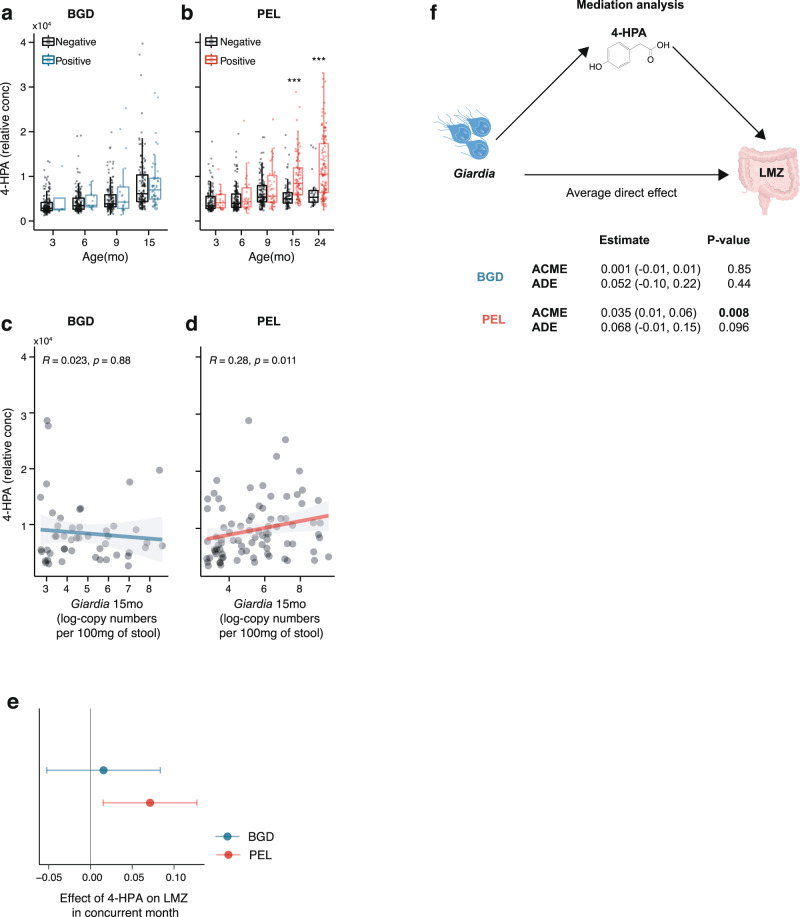
Early childhood *Giardia*-associated gut dysfunction is mediated by specific alterations in byproducts of host-microbial aromatic amino acid metabolism. Relative concentration of urinary 4-hydryxyphenylacetate (4-HPA) (**a**), in Bangladeshi children (3–15 months old) with positive (*N* = 4, 11, 18, 48 for each month respectively) or negative (*N* = 174, 174, 157, 143 for each month respectively) *Giardia* detections (**b**), in Peruvian children (3–24 months old) with positive (*N* = 19, 26, 57, 83, 121 for each month respectively) or negative (139, 134, 286, 83, 32 for each month respectively) *Giardia* detections. (median ± IQR, ***P < 0.001, *P* = 5.4e-10 month 15 and *P* = 1.6e-06 month 24 for *Giardia* negative versus *Giardia* positive in Peru, two-sided Mann-Whitney U-test). **c**, **d** Correlation between urinary 4-HPA and concurrent *Giardia* at 15 months of age in **c**, Bangladesh (*N* = 48) and (**d**), Peru (*N* = 157). Shown are trendlines with 95% confidence interval bands. Two-tailed Spearman’s rank correlation was used to calculate correlation coefficients (R) and *P*-values (p). **e** Effect size of 4-HPA on LMZ in children from Bangladesh (*N* = 210) and Peru (*N* = 193). The mean difference estimate was calculated in a mixed-effects model, adjusted for age, sex, socioeconomic status and *Giardia* quantitative burden. **f** Models were built to assess the degree of the association between *Giardia* infection and LMZ mediated by 4-HPA. Mediation revealed that 4-HPA partially meditates 36% of the effect of *Giardia* on LMZ. Covariates included in the modelling process included sex, age and socioeconomic status. Only children with matching, urinary 4-HPA, LMZ and *Giardia* detection data were included in the analysis. To facilitate comparisons between Peru and Bangladesh only matching data from measurements at 3, 6, 9, and 15 months were included in the models since no urinary measurements were available for Bangladesh at 24 months. All data represent biologically independent samples. Source data are provided as a Source Data file.

*Giardia*-mediated disruptions in protein digestion and absorption could alter peptide availability and amino acid metabolism of co-colonizing intestinal microbiota. Using untargeted ^1^H-NMR based urinary metabolic profiling, we have previously published that *Giardia* infection in Conv-raised PD diet-fed mice increased bacterial-host breakdown of AAs resulting in elevated concentrations of bacterial-derived metabolites, including 4-hydroxyphenylacetate (4-HPA, chemical shift: 6.87 ppm(δ)), a phenol ester bacterial byproduct of tyrosine metabolism and its downstream metabolite, 4-cresol sulfate^13^. Using these same techniques (Supplementary Fig. 10), we found that at 15 months-of-age, *Giardia* infection status associated with altered urinary metabolic profiles, and specifically with increased 4-HPA in Peru children, whereas *Giardia* status among children at Bangladesh at 15 months of age had no measurable effect on urinary metabolic profiles (Fig. 6a, b). Further, relative concentrations of urinary 4-HPA were positively correlated with *Giardia* stool infection burden only in Peru (Fig. 6c, d). Urinary metabolomics (but not serum AAs) were also available in a subset of children in Tanzania at 3 timepoints. Similar to Peru, we found an association between *Giardia* quantitative burden and urinary 4-HPA (Supplementary Fig. 10g–k). These associations between *Giardia* and altered 4-HPA concentrations were specific to *Giardia* and persisted even after controlling for enteropathogen co-infections.

In Peru, where *Giardia* quantity was positively associated with intestinal permeability and urinary excretion of 4-HPA was the highest, we observed a significant positive association between 4-HPA and LMZ in concurrent months (0.12; 0.05, 0.19) (Fig. 6e). Finally, 4-HPA acted as a significant partial mediator in the positive association between *Giardia* and LMZ (Fig. 6f; 36% of mediation *P* = 0.008). In contrast, protein intake in the concurrent month failed to mediate the effect of *Giardia* on LMZ or LAZ (ns), consistent with the experimental findings in wild-type GF mice that protein deficiency alone was insufficient to recapitulate *Giardia*-mediated growth and gut permeability phenotypes when intestinal microbiota were absent (Fig. 2i, n; Fig. 4i, j).

## Discussion

The health of the small intestine sets the course for future childhood development. Impaired growth attainment in the first 2 years of life in children in LMICs associates with heavy and prolonged, yet asymptomatic, exposures to intestinal pathogens like *Giardia*^4^. We report for the first time that *Giardia* associates with dose-dependent disruption of the intestinal barrier and linear growth: greater quantitative exposures enhance the magnitude of small intestinal dysfunction as measured by LMZ and LAZ. Even still, these quantitative-analysis estimates remain site-specific suggesting an additional requirement of parasite^2,37^, host, or in the data presented, environmental (i.e. microbial) factors that mediate *Giardia* pathogenesis. In neither the MAL-ED cohort nor in the murine model do we demonstrate a role for functional evidence of chronic duodenitis in this process. Indeed, accumulating data in experimental models supports that EED as a common final pathway may be an oversimplification of the mechanisms of microbial-mediated gut dysfunction and consequences of disrupted intestinal microbial ecology on early childhood intestinal health. In experimental models, conventional pathogens are not always required to induce EED pathology arising from defined microbial communities^38^. Further, fecal microbiota from children with severe malnutrition can cause rapid wasting in GF mice absent intestinal inflammation^39^. Based on empirical biological plausibility in our novel mono-association model and strong associations in the Peruvian longitudinal birth cohort, we propose an alternative pathway whereby *Giardia* disrupts host-microbiota nutrient metabolism without promoting EED-like intestinal inflammation.

Estimates of *Giardia*-mediated impacts on childhood health in published literature vary, at times showing significant associations with poor weight and/or linear growth gains^3,4,6^, sometimes together with altered gut permeability^8^, and other times measuring no apparent effect^10,11^. There was careful attention to standardization of data collection, pathogen detection, and EED biomarker measurements across all MAL-ED sites. This allowed us the best opportunity to-date to investigate the potential co-factors mediating these outcomes. Our comparison between two sites with divergent associations between *Giardia*, LAZ, and LMZ provides a lens into possible factors that may contribute to *Giardia* and subsequent growth faltering pathogenesis. Interestingly, the divergences in *Giardia-*associated outcomes that are greater in Peru than Bangladesh emerge after cessation of breast feeding when *Giardia* quantitative burden increases convergent with greater EED scores in Peru. A longer duration of exclusive breastfeeding associates with decreased risk of incident *Giardia* detection^3^. Further, the rise in *Giardia* quantity after early weaning in our data herein may indicate a role for constituents in breast milk to limit if not prevent *Giardia*-enteropathy through IgA-^40^ and/or metabolite-mediated^41,42^ inhibition of *Giardia* replication, or by supporting mutualistic intestinal resident microbes and epithelial cell homeostasis. Interestingly, both EED and *Giardia* burden were greater at 15 months-of-age in Peru, despite no apparent difference in other quantitative burdens of other pathogens at that timepoint. Although we do not find associations between *Giardia* and increased EED inflammatory markers, the co-existence of EED and *Giardia* infection in Peru raises the possibility of potential synergies between EED and/or EED-associated microbes, and *Giardia* infection. For example, in our analysis enteroaggregative *E. coli* associated with increased EED composite scores, and we previously published that *Giardia* and EAEC combine to worsen growth outcomes in co-infected mice via separate pathways^13^. Additionally, others have suggested that *Giardia* may enhance virulence of intestinal bacteria^43–45^.

Our combined data suggests that there is an association between host nutritional intake and outcomes following *Giardia* infection. Reductions in serum AAs have previously been associated with stunted growth in Malawi^46^ and Peru^47^. Reduced serum AAs in children with *Giardia* infection could indicate inadequate protein intake, inappropriate balance of peptide absorption and loss through a leaky gut, and/or depletion of amino acids through abnormal host-microbial co-metabolism. Indeed, we have previously shown that increases in byproducts of bacterial aromatic amino acid metabolism, like 4-HPA and other phenolic acids, associate with diminished linear growth in undernourished children in Brazil^48^. In Peru, associations between *Giardia* and intestinal permeability coincided with broad reductions in serum essential amino acids, but fecal A1AT, a marker of protein-losing enteropathy, was not elevated. Rather, these global reductions in AA’s suggest inadequate intake, loss of epithelial cell absorptive function, and/or competition between parasite and host. Protein absorption predominately occurs in the upper small intestine through the function of brush border peptidases and dipeptide and amino acid transporters. *Giardia* is known to consume specific amino acids, such as arginine^49^, and the parasite can also directly damage epithelial cells in vitro, and specifically can impair microvilli with potential consequences for diminished availability of peptidases and surface area for AA absorption even if villus length is preserved^33,50,51^. In immunodeficient mice we show that at high infection burdens, *Giardia* directly harms the host independent from chronic inflammation and activated T cell-mediated pathogenesis seen in other models^31–33^. Data in the *Rag2*^*−/−*^ mono-association model suggests a key role for adequate protein intake to circumvent parasite-mediated effects to maintain gut barrier integrity and host growth. At lower parasite burdens, however, *Giardia* infection restricts host growth and enhances gut permeability in protein-deprived wild-type mice only when intestinal microbiota is present. Future studies are planned to determine the extent to which amino acid deficiency during *Giardia* infection may arise as a result of competition for limited resources or parasite-induced impairment of epithelial cell nutrient uptake.

Using human and murine models, our study raises a possible ‘triple-hit’ model wherein *Giardia* converges with nutritional and microbial factors to alter intestinal microbiota functions resulting in dysregulated nutrient absorption and generation of potentially toxic microbial metabolic byproducts. Experimental models increasingly point to a restructuring of intestinal microbial communities after *Giardia* infection^13,15,44,52,53^, and that *Giardia* may recruit activated CD8^+^ T cells responsive to inflammatory intestinal bacteria^54,55^. However, the consequences of *Giardia*-altered microbial communities on microbial-host intestinal and metabolic functions, is poorly understood. We previously published that *Giardia* infection in protein deprived conventionally raised mice alters 30 metabolites in urinary profiles, including multiple phenolic acids byproducts of bacterial aromatic amino acid metabolism, such as conversion of tyrosine to 4-HPA^13^. 4-HPA was previously identified as a biomarker of small intestinal enteropathies^56^, but to our knowledge, a quantitative relationship between *Giardia* and urinary 4-HPA and the finding that 4-HPA mediates *Giardia* effects on gut permeability in children has not previously been reported. *Giardia* is not known to produce 4-HPA, but *Giardia* and other specific parasites^57^ do produce the metabolic byproduct tyramine, which may serve as a substrate for the generation of 4-HPA by other microbes. In addition, 4-HPA is an intermediate in the conversion of tyrosine to *p*-cresol, a cytotoxic phenolic acid produced by bacterial tyrosine phenol-lyase (β-tyrosinase)^58^. Identifying the dietary and host-microbial sources that are generating 4-HPA, whether an interaction exists between *Giardia* and other disruptions in small intestinal ecology like small intestinal bacterial overgrowth, and determining if there are direct consequences of 4-HPA on intestinal function require additional study.

We have identified decreased serum AAs and increased AA byproducts like 4-HPA as more promising biomarkers of *Giardia-*mediated enteropathy than current inflammation-targeted EED-based markers. Our study is limited, however, in having a complete set of AAs and metabolomics profiles available from only two of the eight MAL-ED sites. Consequently, we may have been underpowered to identify biologically relevant differences in metabolites other than 4-HPA. Also, future studies will be needed to validate this finding, to address generalizability across multiple different geographic settings and human populations, and to reconcile what other factors (including *Giardia* strain differences beyond the assemblage-level designation, microbiota, and specific dietary components) may drive *Giardia* pathology given the lack of association between *Giardia* and 4-HPA in Bangladesh. Also, urinary ^1^H NMR spectra are not optimized to resolve differences in other important metabolic pathways, such as bile acids and biliary lipids that are altered in some experimental models of early life *Giardia* infection^13,15^. Additionally, while our statistical modelling demonstrated that 4-HPA mediates *Giardia* effects on intestinal permeability, this finding needs to be directly studied and validated in both human cohorts and experimental models. We also did not have access to intestinal samples to perform microbiome-wide analyses nor for the purposes of transferring microbiota from the child cohorts to conventionalize the germ-free mice. Future studies are needed to identify the microbial sources of 4-HPA generation^59^, their regionalization in the gut in respect to *Giardia*, and the functional consequences of interactions between dietary restriction, *Giardia* specific strains, and specific bacteria in the intestinal microbial community.

In conclusion, although *Giardia* is conventionally categorized with diarrheal diseases, we find in childhood cohorts in resource limited settings and in our novel murine mono-association malnutrition models, that *Giardia* primarily disrupts nutrient homeostasis even in the absence of diarrhea. Unlike most bacterial intestinal pathogens that drive inflammation-associated gut dysfunction, absent any apparent provocation of host inflammatory responses in this study, *Giardia* appears to capitalize on its ecological advantages when protein is scarce. Data in this study suggests that in certain environments, *Giardia* and intestinal microbiota may be co-conspirators: diminishing amino acids and fueling potential toxic amino acid byproducts. Thus, we propose that a triple-hit convergence of an intestinal microbial ecology permissive of *Giardia* infection together with limited protein erodes intestinal nutrient-metabolic homeostasis to restrict child growth in diet marginal populations.

## Methods

This study involves human participants. It includes the use of archived deidentified data collected in the MAL-ED study. For the parent study, ethical approval was obtained from the institutional review boards at the University of Virginia School of Medicine (Charlottesville, VA) (14595), and at each of the participating research sites: Ethical Review Committee, International Centre for Diarrhoeal Disease Research, Bangladesh (Bangladesh); Committee for Ethics in Research, Universidade Federal do Ceara, and National Ethical Research Committee, Health Ministry, Council of National Health (Brazil); Institutional Review Board, Christian Medical College, Vellore, and Health Ministry Screening Committee, Indian Council of Medical Research (India); Institutional Review Board, Institute of Medicine, Tribhuvan University, Ethical Review Board, Nepal Health Research Council, and Institutional Review Board, Walter Reed Army Institute of Research (Nepal); Institutional Review Board, Johns Hopkins University, and PRISMA Ethics Committee; Health Ministry, Loreto (Peru); Ethical Review Committee, Aga Khan University (Pakistan); Health, Safety and Research Ethics Committee, University of Venda, and Department of Health and Social Development, Limpopo Provincial Government (South Africa); and Medical Research Coordinating Committee, National Institute for Medical Research, and Chief Medical Officer, Ministry of Health and Social Welfare (Tanzania). The study was done in accordance with the principles stated in the Declaration of Helsinki and Good Clinical Practice guidelines. Signed informed consent was obtained by child guardians for each child participant. No monetary compensation was provided for participation. Deidentified participant data from the MAL-ED study is publicly available at ClinEpiDB.org after approval of a proposal by the study’s principal investigators (PIs).

This study also includes the use of mice. Mouse experiments were conducted in strict accordance with recommendations in the Guide for the Care and Use of Laboratory Animals of the National Institutes of Health. The mouse experimental protocol was approved by the Institutional Animal Care and Use Committee at the University of North Carolina at Chapel Hill (IACUC protocol #18-266).

### MAL-ED Study design, sample collection, processing, and analyte detection

Our study used deidentified participant demographics data that was individually-linked to biologically assay results archived as part of the Etiology, Risk Factors, and Interactions of Enteric Infections and Malnutrition and the Consequences for Child Health (MAL-ED). Using a prospective, longitudinal design, MAL-ED investigated the complex relationship between nutrition and repeated enteric infection on early childhood developmental outcomes, in infant cohorts across eight countries with high burdens of undernutrition and diarrheal disease. Detailed information on the parent study design and the methods used for data collection have been previously published^22^. MAL-ED enrolled both males and females with sex designations as reported by child participant guardians. Methods relevant to the original sample collections and biospecimen processing are summarized below.

#### MAL-ED Sample Collections

Children were enrolled within 17 days of birth and followed for 24 months. Stool samples were collected monthly in the absence of diarrhea from all children in the MAL-ED cohort (*n* = 2134 children). Raw stool aliquots were stored at −80 °C before total nucleic acid extraction^22^.

For the present study, plasma amino acids and urinary metabolite profiles were acquired from plasma and urine samples collected from two cohorts in MAL-ED: Peru and Bangladesh. Serial urine samples were collected at the 3, 6, 9, 15, and 24 months for Peru (*n* = 281 children) and at 3, 6, 9, and 15 months for Bangladesh (*n* = 249 children). The urine sample set for Peru included 1057 samples and 860 for Bangladesh. Plasma samples were collected from fasted children in Peru (*n* = 141) and Bangladesh (*n* = 193) at 15 months of age. Serial urine samples (but not plasma) were also collected at 6, 15, and 24 months from children in Tanzania (*n* = 164). Samples were stored at −80 °C and shipped in dry ice to Imperial College London for analysis.

For lactulose:mannitol (L:M) testing, children at 3, 6, 9, and 15 months of age, were fasted for a minimum of 2 h before (with the exception of breast milk which was permitted *ad libitum*), and 30 min following the administration of the lactulose/mannitol solution. The solution included 250 mg/mL of lactulose and 50 mg/mL of mannitol (1002 mOsm/L), at a dose of 2 mL/kg up to a maximum of 20 mL. Urine samples were collected using a urine collection bag that was placed and changed as needed for the following 5-hour period. Samples were aliquoted and stored at −70 °C before testing. Additional details have been previously described in ref. ^60^.

#### Quantitative Giardia detection from stool samples

Total nucleic acid was extracted from stool samples using the QIAmp Fast DNA Stool Mini Kit (Qiagen). For the subset of 1715 children with complete follow-up, all non-diarrheal stools (*N* = 34,614) were tested by qPCR for *Giardia* (specifically, the 18 S rRNA gene) using custom designed TaqMan Array Cards (Thermo Fisher, Carlsbad, CA, USA). All laboratory methods, including assay validation and quality control, have been described in refs. ^61,62^. Detections at a cycle threshold value of less than 35 were considered positive for *Giardia*. *Giardia* quantities were based on the cycle threshold and described as mean log-copy numbers per g of stool. Non-diarrheal stool samples collected monthly in the first year of life and quarterly in the second year of life from all 2134 children in the MAL-ED cohort were tested for EED biomarkers: alpha-1-antitrypsin (AAT), myeloperoxidase (MPO), and neopterin (NEO) as previously described in ref. ^63^.

#### Lactulose:Mannitol assays

Detailed description of the methodology used for lactulose:mannitol (L:M) assays, the calculation of lactulose:mannitol ratios, as well as the process followed for age and sex normalization are reported Kosek et al. 2017 ^60^. Briefly the L:M ratio = % lactulose recovered / %mannitol. % lactulose recovered = urine concentration lactulose [mg/L] × urine volume [L] × 100/total lactulose dosed [mg] and % mannitol recovered = urine concentration mannitol [mg/L] × urine volume [L] × 100/total mannitol dosed [mg]. Ratios were converted into sample-based z scores (LMZs) using the MAL-ED cohort in Brazil as the internal reference population as previously described in ref. ^60^.

#### Diet data collection and handling

At twice-weekly home visits, caregivers were asked about feeding practices on the previous day, as described previously in refs. ^24,64^. Breastfeeding practices were categorized as exclusive, predominant (received only breast milk or breast milk with water or clear liquids), partial (received breast milk plus other milk or food) or none. Reported feeding patterns were assumed to remain the same until the next available surveillance visit data. At 9 months of age and monthly thereafter, 24 h recalls were conducted and linked to food composition databases that were specific to each site to estimate energy, macronutrient, and micronutrient intakes from non-breast milk foods. The mean energy in kcal from protein was calculated for each child. In the data presented, complete dietary data was available for 229 of 303 children enrolled in Peru and for 233 of 265 children enrolled in Bangladesh.

#### ^1^H NMR spectroscopy and UPLC-MS based metabolic profiling in children

Urinary metabolic profiles were measured by ^1^H NMR spectroscopy using the protocols described by Dona et al.^65^ and Beckonert et al.^66^. Briefly, 630 μl of urine sample were combined with 70 μl of phosphate buffer solution (pH 7.4, 100% D_2_O) containing 1 mM of the internal standard, 3-trimethylsilyl-1-[2,2,3,3-2H4] propionate (TSP). Samples were then vortexed and spun (12,000 g) for 10 min at 4 °C before transfer to 5 mm NMR tubes. A pooled urine quality control (QC) sample was prepared by combining 5 μl of each individual sample of the study and was used to monitor variability of the analytical platform. One dimensional 600 MHz ^1^H NMR spectra were acquired on a Bruker NMR spectrometer (Bruker Biospin GmbH, Rheinstetten, Germany), equipped with a SampleJet system and a cooling rack of refrigerated tubes at 6 °C. ^1^H NMR spectra acquisition was achieved using a standard one-dimensional solvent suppression pulse sequence (relaxation delay, 90° pulse, 4 μs delay, 90° pulse, mixing time, 90° pulse, acquire FID). For each sample, 32 transients were collected in 64 K frequency domain points with a spectral window set to 20 ppm. A relaxation delay of 4 s, a mixing time of 10 ms, an acquisition time of 2.73 s and 0.3 Hz line broadening was used. Spectra were referenced to the TSP resonance at δ 0.0. Spectral phasing and baseline correction were automatically performed using Topspin 3.2 (Bruker Biospin GmbH, Rheinstetten, Germany). The resulting raw NMR spectra were digitized, aligned and normalized using the Imperial Metabolic Profiling and Chemometrics Toolbox (https://github.com/csmsoftware/IMPaCTS) in MATLAB (Version 2018a, Mathworks Inc). Briefly, after digitization of the spectra, redundant peaks (TSP, H_2_O and urea) were removed and the resulting spectra were manually aligned to reference peaks using Recursive Segment-Wise Peak Alignment^67^. The aligned spectra were normalized using probabilistic quotient normalization^67^. This approach adjusts the metabolite concentrations for differences in sample dilution as a result of differences in liquid and food intakes between infants, offsetting any potential confounding effect from these factors^68^. The relative concentration of 4-hydroxyphenylacetate was calculated from the spectral data using trapezoidal numerical integration.

A targeted amino acid assay was carried out using UPLC-MS for the plasma samples using a Waters Acquity UPLC coupled to a XEVO TQ-XS mass spectrometer following the method published by Gray et al.^69^. For the plasma samples, data were acquired and processed using Waters MassLynx (Version 4.2). All statistical analyses were performed using RStudio (Version 1.2.1335). Statistical tests used are stated in the figure captions. Differences of *p* < 0.05 were considered significant.

### Animals and animal handling procedures

All experiments used *Mus musculus* research mice. Mice were housed in light cycles of 12:12 (12 h light and 12 h dark), at the set temperature of 72 °F ( ± 3 °F) and a set humidity range of 30–70%. All cages, food, water, and bedding were autoclaved prior to use.

For specific pathogen free (SPF) mice, all experiments used male wild-type C57Bl/6 J mice obtained from Jackson Laboratories. SPF mice were age-matched at 21 days of life and at least 10 g prior to shipping. Mice acclimated immediately on respective diets. Mice were housed 1–2 mice/cage in individually ventilated cages (IVCs) in the Division of Comparative Medicine BSL2 isolation cubicle facility at UNC-CH. Male mice were used for SPF experiments based on previous publications of experimental *Giardia* infection frequently using only male mice.

For experiments in germ-free (GF) mice, all experiments used both male and female C57Bl/6 J wild-type and *Rag2*^*−/−*^ (C57Bl/6 J background) mice at 8–16 weeks-old obtained from the National Gnotobiotic Rodent Resource Center at UNC-CH. For each experiment, mice were sorted into age and sex-matched intervention and control groups and co-housed 3–5 per cage in GF Trexlar isolators or 1–2 per cage if transferred from isolators to the SPF cubicle facility. Mouse weights were obtained using a battery-operated digital scale (Ohaus) with a precision of +/− 0.01 g. For use in GF isolators, the scale and batteries were sterilized using ethelyne oxide at Andersen, Scientific (Raleigh, NC). Fecal pellets were obtained as previously described in ref. ^70^. Additional details of individual mouse sex, age, and caging for individual experiments are provided in the Source Data file.

### Mouse diets

For all SPF experiments, mice were fed either the Protein deficient diet (Envigo, TD.110200) or the isocaloric control diets (Envigo, TD.08678). These diets are casein-based diets that are certified according to manufacturer standards. Since casein-based diets may contain lactic acid bacteria, the protein deficient diet was autoclaved prior to use in GF isolators to ensure sterility. The custom isocaloric control diet did not withstand autoclave conditions. Therefore, for germ-free experiments, the germ-free mice in isolators were fed the standard NGRRC autoclaved chow (Envigo, 2020SX) as a control diet.

### Giardia cyst preparation and sterility testing

Commercial *G. lamblia* (assemblage B, H3) cysts were purchased from Waterborne, Inc (New Orleans, LA). Cysts were prepared at Waterborne, Inc. after passage through gerbils and purified according to on-site protocols. Confirmatory assemblage typing was previously performed^12^. For identification of microbial contaminants in the commercial cyst preparation, unprocessed gerbil stool and purified *Giardia* cysts were cultured at UNC-CH using Luria-Bertani (LB), Sheep’s Blood, Brain Heart Infusion, and Sabouraud dextrose agar (Remel) for 7 days, and liquid Thioglycolate (Remel) for 48 h at 37 °C. Bacterial isolates were sub-cultured onto LB agar plates and identified by matrix assisted laser desorption ionization-time of flight mass spectrometry (MALDI-TOF MS) and yeasts were identified by whole-genome sequencing using clinical reference databases in the UNC-CH McClendon Laboratory (Supplementary Fig. 6). Ultra-pure cysts were generated at Waterborne, Inc. by adding the following steps to the routine protocol: sterile-filtering all solutions used in the purification process, rinsing cysts with Tween 80, performing a second percoll gradient (at 1.07 g/mL and 1.09 g/mL), and resuspending cysts in sterile-filtered standard preservation solution supplemented with ampicillin (1000 *μ*g/mL) (Sigma-Aldrich) to deplete gram-positives and micafungin (1000 *μ*g/mL) (Sigma-Aldrich, SML2268) to deplete bleach-tolerant *Candida* spp. (Supplementary Fig. 6). Ultra-pure cysts were shipped overnight and immediately rinsed in PBS upon receipt at UNC-CH. Limiting dilutions identified a lower limit of 10^2^ ultra-pure cysts to establish infection in *Rag2*^*−/−*^ mice without evidence of another microbe (Supplementary Fig. 6). When using commercially-derived H3 cysts for experimental challenge, challenge inoculums were determined by first culturing the cyst preparation and challenging mice with the highest concentration of cysts (10^3^−10^5^ depending upon the lot) that resulted in no growth of bacteria at 48 h incubation. The challenge dose for each experiment is indicated in the respective figure legends.

### Giardia propagation and axenic cyst challenges

To establish axenic *Giardia* mono-association propagation in GF mice, 10^2^ ultra-pure commercial *G. lamblia* H3 cysts (Waterborne, Inc) were inoculated into 2 individually caged GF *Rag2*^*−/−*^ mice. One of these mice developed *Giardia* mono-associated infection and became the founder for all subsequent propagations (Supplementary Fig. 7). *Giardia* was transmissible in GF isolators only by direct inoculation (Supplementary Fig. 7). For challenge experiments in GF isolators, 10–15 fresh fecal pellets were collected from a single mouse and suspended in 5 mL PBS (400–500 *μ*l per pellet). The pellets were homogenized with sterile wooden swabs and mixed by manual inversion and agitation. The slurry was allowed to sediment by gravity for 2 min prior to removing the supernatant. Each mouse was inoculated with 200 *μ*l of slurry by orogastric gavage. The concentration of cysts for each challenge experiment was verified on the day of challenge (10^3^/100 *μ*l) using the remnant slurry. For challenge experiments in SPF gnotobiotic barrier facility cubicles, 10^3^/100 *μ*l axenic cysts were similarly prepared from colon contents of a single GF propagator removed from the isolator on the day of challenge.

### Fecal microbiota transplant

To transfer fecal microbiota from SPF CD-fed or PD-fed uninfected mice, fresh fecal pellets were collected serially between 7 and 21 days on diet. The pellets were then snap-frozen and stored at −80 °C. On the day of challenge, frozen fecal pellets were pooled, thawed, diluted in PBS (10 mg/mL of PBS), and homogenized. Mice were inoculated with 100 *μ*l of the homogenate by orogastric gavage according to experimental timelines (Supplementary Fig. 4).

### In vivo Intestinal permeability

Mice were fasted for ≥ 4 h prior to planned serum collection. On the day of serum collection 4.4 kDa FITC-dextran (Sigma-Aldrich, 46944) in PBS at a concentration of 200 mg/ml (wrapped in foil to avoid light) was administered by oro-gastric gavage (125 mcl). Gavage timing was staggered to ensure that cardiac puncture after anesthesia occurred 1.5 h after oral gavage. SPF mice were euthanized in two batches with equal representation of all groups (*N* = 3/group) in each batch. Concentrations of controls and serum samples were determined by spectrophotometry with an excitation of 485 nm (20 nm band width) and an emission wavelength of 528 nm (20 nm band width).

### Giardia detection in mice

*Giardia* trophozoites and cysts were detected by light microscopy and enumerated with a hemocytometer. Small intestinal fragments of 4 cm in length were taken 1 cm from the pyloric sphincter (duodenum), 1 cm from the cecum (ileum), and mid-intestine (jejunum) and then opened longitudinally, minced, and placed in 4 mL of ice-cold PBS for 30–45 min as previously described in ref. ^13^. Trophozoites were counted in 10 *μ*l aliquots using a hemocytometer and the final count represented an average of 2 readings per fragment. If trophozoites were not detected by this method (i.e., uninfected controls), the segment was transferred to a 12-well culture plate and samples were examined for at least 15 min per sample. Fecal cysts were also detected by suspending fecal pellets into 200–400 hemacytometer of PBS and visualized using hemocytometer. Immunofluorescent staining of *Giardia* cysts (Giardi-a-Glo®, Waterborne, Inc.) was performed according to manufacturer instructions^12^. Immunofluorescence images were captured on an Olympus 1X 71S8F-3 microscope at 100X200X/400X magnification with an Olympus U-LH100HG light source and DP27 camera using cellSens Life Science imaging software.

### Histology

Longitudinal and cross-sectional sections from 2–4 cm segments of duodenum, jejunum, and ileum were prepared as previously described in ref. ^13^. Briefly, sections were placed in 10% Zn-formalin x 48 h before transfer into 70% ethanol. Sections were paraffin-embedded, sectioned, and stained in the Center for Gastrointestinal Biology and Disease Histology Core. Morphometry was measured using image J on live-images captured using Olypmus cellSens Standard 1.14 software and an Olympus 1 × 71 microscope. Measurements were performed by (VT) who was blinded to experimental groups. Independent histological review was performed in the UNC Tissue Pathology Laboratory (SM) who was also blinded to treatment groups. Photomicrographs were captured on an Olympus BX43 light microscope at 100X200X/400X magnification with a DP27 camera using cellSens Entry software.

### Targeted amino acid quantification—mice

Blood was collected by cardiac puncture at the time of mouse necropsy, and serum was separated using BD (365967) serum separator additive microtainer tubes and centrifugation at 4000 x g rcf for 15 min. The recovered Serum was stored at −80 °C. Fecal samples also collected at the time of sacrifice were snap-frozen in liquid nitrogen prior to storage at −80 °C. All mouse were samples shipped on dry ice to the University of Pennsylvania Microbial Culture and Metabolomics Core. The Core performed targeted amino acids quantification using a Waters Acquity uPLC System with a Photodiode Array Detector. Amino acid concentrations are quantified via ultra-performance liquid chromatography (Waters Acquity UPLC system) with an AccQ-Tag Ultra C18 1.7 μm 2.1 x 100 mm column and a photodiode detector array. Analysis was performed using the UPLC AAA H-Class Application Kit (Waters Corporation, Milford, MA) according to manufacturer’s instructions. Standards are run at the beginning and end of each metabolomics run. Quality control checks (blanks and standards) are run every eight samples. Results are rejected if the standards deviate by greater than ± 5%. All chemicals and reagents are mass spectrometry grade. Samples without detectable analytes were assigned a value at limit of detection, which is 1 nmol/g in stool or 3 uM in plasma.

### Statistics and reproducibility

#### Human cohort (MAL-ED)

The present study used all data accessible through the MAL-ED archived de-identified data set. The original MAL-ED protocol targeted enrollment to > 200 children per site. These sample sizes were established in the original MAL-ED protocol, and we therefore used all de-identified data available without any additional statistical method to predetermine sample size for the analysis in the present study. No data were excluded from these analyses. The investigators were not blinded to outcome assessment*.*

### Growth and EED associations

In 1715 children in the MAL-ED cohort, we estimated the association between average *Giardia* quantity in non-diarrheal stools collected monthly from 0–23 months of age and LAZ at 2 years using linear regression and adjusting for site and for parameters that influence LAZ at 2 years of age as previously in ref. ^4^: enrollment LAZ, sex, SES, exclusive breastfeeding duration in the first 6 months of life and maternal height. *Giardia*-negative stools were included using a value of half the limit of detection, and effects were scaled per 2-log increase in average *Giardia* quantity. In a sensitivity analysis, the model was further adjusted for average monthly quantitative burden of other pathogens previously associated with LAZ at 2 years of age in MAL-ED: *Campylobacter*, *Shigella*, enteroaggregative *E. coli* (EAEC), and *E. bieneusi*^4^.

We estimated associations between *Giardia* detection in non-diarrheal stool samples and lactulose:mannitol z-score (LMZ) measured in urine at the same month of age using linear regression with generalized estimating equations to account for repeated measurements within children and adjusting for site, age, sex, SES, and exclusive breast-feeding duration in the first 6 months of life. Similar models were used to estimate the association between *Giardia* quantity and LMZ, with effects scaled per 2-log increase in *Giardia* quantity. In sensitivity analyses, we additionally adjusted for the presence of other pathogens in the same stool sample associated with increased LMZ (*p* < 0.05), including *E bieneusi*, *Cryptosporidium*, atypical EPEC, norovirus, astrovirus, and sapovirus. We used similar models to estimate associations between *Giardia* quantity and neopterin, myeloperoxidase, and alpha-1-antitrypsin concentrations in the same stool sample, additionally adjusting for stool consistency which can affect stool biomarker concentrations. In sensitivity analyses, we additionally adjusted the model for the presence of other pathogens in the same stool sample associated with each biomarker outcome (*p* < 0.05). This included *Shigella*, *Cryptosporidium*, *Campylobacter*, EAEC, typical EPEC, atypical EPIC, norovirus, ETEC, and *E bieneusi* for myeloperoxidase; typical EPEC, norovirus and adenovirus for alpha-1-antitrypsin; and *Cryptosporidium* for neopterin.

We additionally estimated associations between *Giardia* quantity and components of the EED score: myeloperoxidase, neopterin, and alpha-1-antitrypsin concentrations in stool samples collected at 6, 15, and 24 months of age in Bangladesh and Peru using linear regression and adjusting for site, sex, SES, stool consistency, and exclusive breast-feeding duration in the first 6 months of life. Samples without *Giardia* were similarly included at half the limit of detection and effects were scaled per 2-log increase in *Giardia* quantity. In all models, site-specific estimates were derived by including an interaction term between *Giardia* exposure variable and site.

EED biomarker score for each child was calculated using the following equation as described previously in ref. ^38^. All analyses were performed in SAS version 9.4 and all the models were run using PROC GENMOD package.

### Breastfeeding practices analysis

To determine the duration of any breastfeeding, breastfeeding logs from Bangladesh and Peru were filtered for age on the last recorded date of breastfeeding (e.g., exclusive, predominate, or partially breastfed). For cessation of exclusive breastfeeding dietary logs were filtered for age on the last recorded date of exclusive breast feeding. If breastfeeding status was not recorded, data were excluded from the analysis. Median values and confidence intervals were determined using R 4.0.2 (Vienna, Austria), all tests were two sided, and a *P*-value of < 0.05 was considered statistically significant.

#### Giardia-metabolite associations

Orthogonal projections to latent structures regression (OPLS) models were constructed to identify urinary metabolic features associated with *Giardia* infection, where the ^1^H NMR metabolic profiles served as the descriptor matrix and the quantitative burden of the pathogen was the response variable. *Giardia* discriminant features were identified using in-house databases and the Human Metabolome Database (http://www.hmdb.ca/). The predictive power of each model was calculated using sevenfold cross-validation approach, and model validity (presented as P value) was calculated by permutation testing (1000 permutations; significance for Pper <0.05). Models were built using MATLAB (Version 2018a, Mathworks Inc.) and scripts from the Imperial Metabolic Profiling and Chemometrix Toolbox (https://github.com/csmsoftware/IMPaCTS).

The two-sided Mann-Whitney U-tests test was used for comparisons of metabolite levels between *Giardia* positive and negative children at each age. Spearman’s correlations were used to assess the relationship between 4-HPA levels and *Giardia* quantitative burden. All statistical analyses were performed using R 4.0.2 (Vienna, Austria), all tests were two sided, and a *P*-value of < 0.05 was considered statistically significant.

We estimated associations between 4-HPA and LMZ in urine samples collected at 3, 6, 9, and 15 months of age in Bangladesh and Peru using linear regression (lme4 package in R) and adjusting for age, sex, SES and *Giardia* quantity. To formally assess the contribution of 4-HPA to the association between *Giardia* and gut permeability, mediation analysis was conducted using the ‘mediation’ package in R. 4-HPA was considered as the mediator for the effect of the pathogen on LMZ. Linear mixed effects models were used for both the mediator and outcome models. The average causal mediation effect (ACME) and the average direct effect (ADE) are reported for both Bangladesh and Peru models.

### Mouse model data

#### General

For all murine experiments, animals were randomized into weight-matched groups at baseline. Due to the requirements to label infectious agents, the investigators were not blinded to allocation during experiments or to growth outcomes*.* A deidentification method was used to blind investigators to outcome assessments of *Giardia* status by live microscopy, histopathology analysis, and serum FITC measurements. No statistical method was used to predetermine sample size. Animal weight data were normalized to baseline absolute weights on day of challenge. For all growth assessments the absolute weights were transformed as percent of initial weight were analyzed using a two-way ANOVA test with repeated measures and Bonferroni post-test analysis for multiple comparisons. For comparisons between only two groups normalized data was compared using two-sided Unpaired t-tests and for non-normalized data two-sided Mann-Whitney U-test was used. The One-Way ANOVA with Holm-Sidak’s or Kruskal-Wallis with Dunn’s multiple comparisons correction tests were used for multi-group comparisons for normalized and non-normalized data, respectively. Differences were considered significant at *P* < 0.05. Analyses for all data obtained from mouse models were analyzed with GraphPad Prism version 9.3. Data from all independent experiments are shown in the main figure or in the supplementary figures. Every datapoint indicates measured performed on independent biological replicates as either a single measurement or the mean of technical replicates.

### Reporting summary

Further information on research design is available in the Nature Portfolio Reporting Summary linked to this article.

## Supplementary information

Supplementary Information

Reporting Summary

## Data Availability

The metabolomics data generated in this study were identified using the Human Metabolome Database and have been deposited on Zenodo under the access link 10.5281/zenodo.7678773. All aggregate source data for the results reported in this study are provided in the Source Data file. Due to data ownership requirements, we are not permitted to provide disaggregated data from MAL-ED, but individually-linked data underlying the results presented in the study are available from the ClinEpiDB database (https://clinepidb.org/ce/app/workspace/analyses/DS_5c41b87221/new/details). One must register and request access to download data; data can be downloaded after a committee reviews the request and grants access. All disaggregated individually-linked raw data for all mouse experimentation are present in the Source Data file. Source data are provided with this paper.

## Source data

Source Data

## Code availability

All technical aspects related to the analysis of the data used existing packages and software that are fully detailed in the Methods section.
