# Supplementary material for: Giardia hinders growth by disrupting nutrient metabolism independent of inflammatory enteropathy
Source: Nat Commun. 2023 May 18;14:2840. doi: 10.1038/s41467-023-38363-2 (PMC10195804; doi:10.1038/s41467-023-38363-2)
Supplement: Supplementary file 1 — Supplementary Information [file 41467_2023_38363_MOESM1_ESM.pdf]

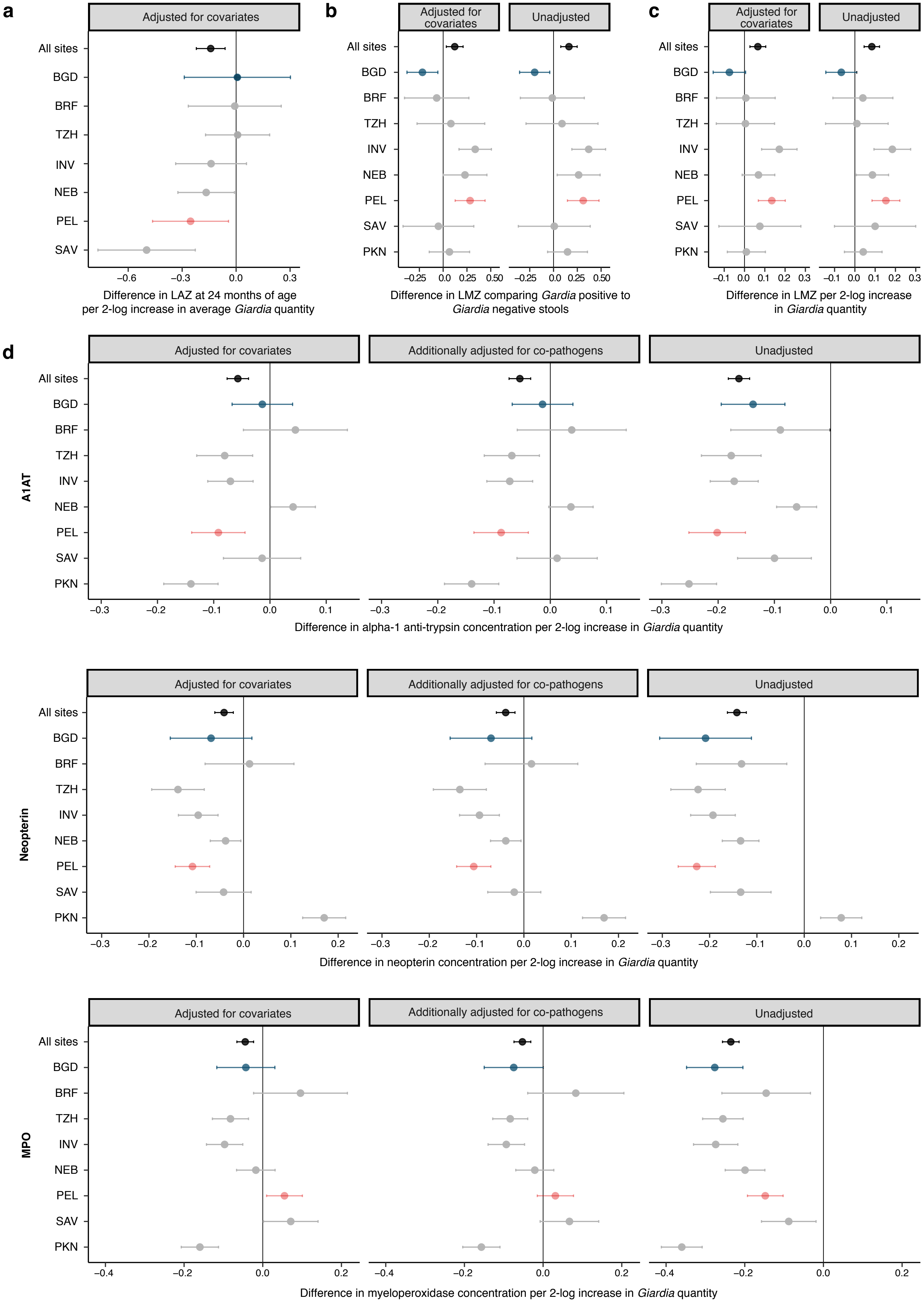

a
Adjusted for covariates
All sites
BGD
BRF
TZH
INV
NEB
PEL
SAV
−0.6
−0.3
0.0
0.3
Difference in LAZ at 24 months of age
per 2-log increase in average Giardia quantity
b
Adjusted for covariates
Unadjusted
−0.25
0.0
0.25
0.50
PKN
Difference in LMZ comparing Gardia positive to
Giardia negative stools
c
−0.1
0.1
0.2
0.3
Difference in LMZ per 2-log increase
in Giardia quantity
d
Additionally adjusted for co-pathogens
A1AT
Difference in alpha-1 anti-trypsin concentration per 2-log increase in Giardia quantity
Neopterin
Difference in neopterin concentration per 2-log increase in Giardia quantity
MPO
Difference in myeloperoxidase concentration per 2-log increase in Giardia quantity

Supplementary Figure 1. Associations between *Giardia* status and quantity with linear growth and EED biomarkers, aggregate and site-specific.

a, Association between *Giardia* detection and LAZ across all MAL-ED sites. Estimates presented are adjusted for enrolment LAZ, sex, socioeconomic status, maternal height and exclusive breastfeeding in the first 6 months of life. Mean difference point estimate and 95% confidence intervals shown for: **a)** All sites (*N*=1469 individual participants), Bangladesh (BGD; *N*=210), Peru (PEL; *N*=194), Brazil (BRF; *N*=165), India (INV; *N*=227), Nepal (NEB; *N*=227), Pakistan (PKN; *N*=246), South Africa (SAV; *N*=237), Tanzania (TZH; *N*=209); **b,** *Giardia* detection and **c,** *Giardia* quantity and LMZ across all MAL-ED sites. Mean difference point estimate and 95% confidence intervals shown for: All sites (N=3160 biologically independent measurements); BGD (*N*=525); PEL (*N*=337); BRF (*N*=229); INV (*N*=529); NEB (*N*=601); PKN (*N*=393); SAV (*N*=345); TZH (*N*=201)**.** Data presented on the left panels are adjusted for age, sex, socioeconomic status, and exclusive breast feeding in the first six months of life without additional adjustment for *Enterocytozoon bieneusi, Cryptosporidium,* atypical enteropathogenic *E coli*, Norovirus, Astrovirus and Sapovirus. Unadjusted estimates are shown on the right panel. **d,** Association between alpha-1-anti-trypsin (top row), neopterin (middle row), and myeloperoxidase (bottom row) concentration and *Giardia* quantity, across all MAL-ED sites. Estimates presented on the left panels are adjusted for age, sex, socioeconomic status, exclusive breast feeding in the first six months, and stool consistency. Mean difference point estimate and 95% confidence intervals shown for: All sites (N=19,009), BGD (*N*=2574); PEL (*N*=2114); BRF (*N*=1500); INV (*N*=2782); NEB (*N*=2888); PKN (*N*=2401); SAV (*N*=2560); TZH (*N*=2190) biologically independent measurements for each target. Data presented on the left panels are adjusted for age, sex, socioeconomic status, and exclusive breast feeding

in the first six months of life. Data on the middle panels are additionally adjusted typical EPEC, norovirus, and adenovirus (alpha-1-anti-trypsin, top row); for *Cryptosporidium* (neopterin, middle row); and for *Shigella, Cryptosporidium, Campylobacter,* EAEC, typical EPEC, atypical EPEC, norovirus, ETEC and *E bieneusi* (myeloperoxidase, bottom row). Unadjusted estimates are shown on the right panel. All estimates were calculated using generalized estimating equations. All data represent biologically independent samples. Source data are provided as a Source Data file.

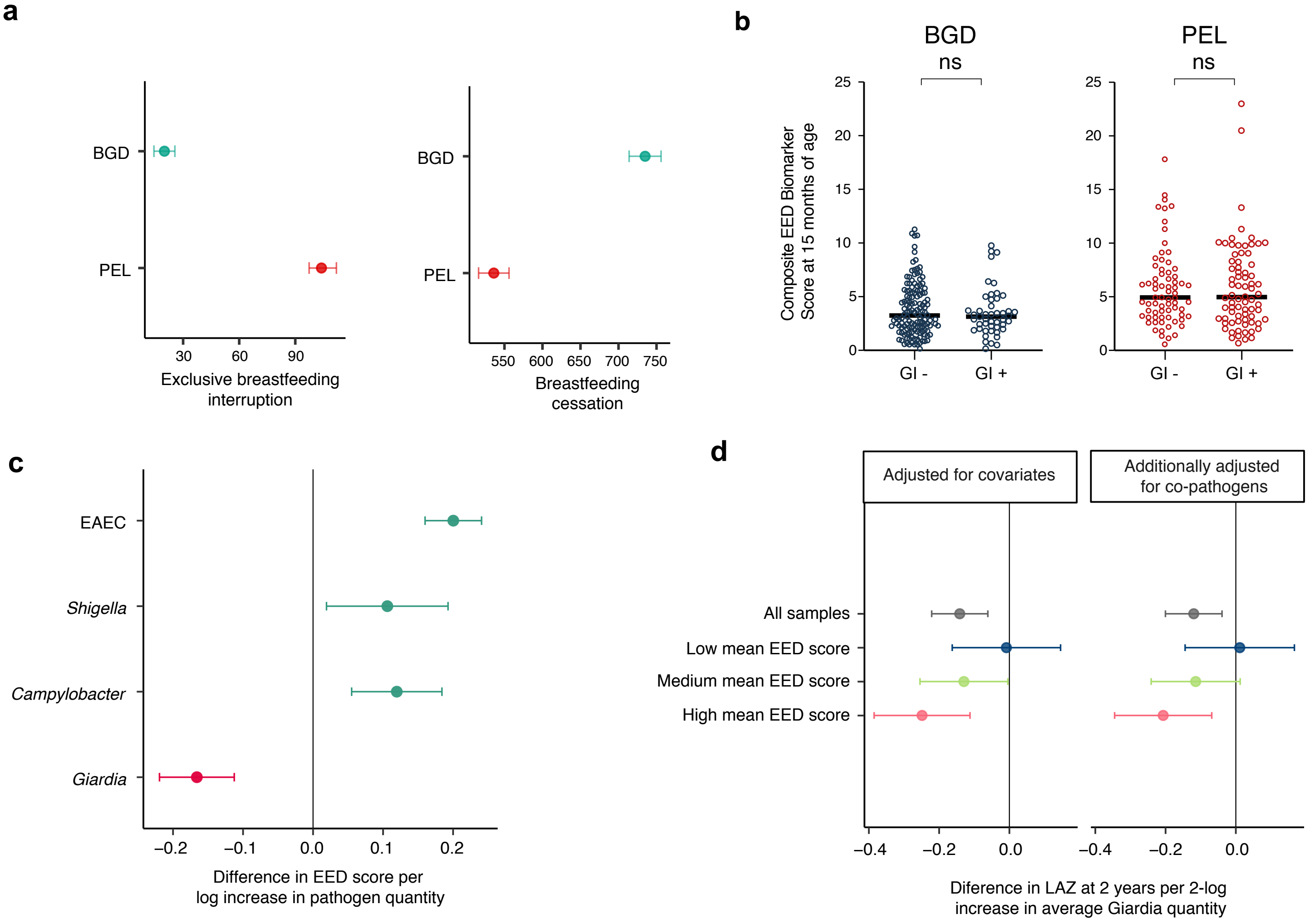
a
BGD
PEL
30
60
90
Exclusive breastfeeding interruption
BGD
PEL
550
600
650
700
750
Breastfeeding cessation
b
BGD
ns
PEL
ns
Composite EED Biomarker Score at 15 months of age
25
20
15
10
5
0
GI -
GI +
c
EAEC
Shigella
Campylobacter
Giardia
−0.2
−0.1
0.0
0.1
0.2
Difference in EED score per log increase in pathogen quantity
d
Adjusted for covariates
Additionally adjusted for co-pathogens
All samples
Low mean EED score
Medium mean EED score
High mean EED score
−0.4
−0.2
0.0
Diference in LAZ at 2 years per 2-log increase in average Giardia quantity

Supplementary Figure 2. Associations between specific pathogens and EED fecal biomarkers

a, Breastfeeding patterns in Bangladesh (BGD; N=248) and Peru (PEL; N=281) from birth to 24 months of age. left, interruption of exclusive breastfeeding; right, cessation of any breast feeding including exclusive, predominant, or partial breastfeeding. Median point estimates and confidence intervals (P<0.0001 for both comparisons, two-sided Mann-Whitney U-test).

b, EED scores in BGD (N=183) and PEL (145) at 15 months in children with (N=47 in BGD, N=74 in PEL) or without *Giardia* (N=136 in BGD, N=71 in PEL) present in their stool (median ± IQR, two-sided using Mann-Whitney U-test (ns, P>0.05). **c,** Association between EED score and EAEC, *Shigella, Campylobacter* and *Giardia* quantity, across all MAL-ED sites (N=1714). Shown as median difference and 95% confidence interval. Estimates presented are adjusted for age, sex, socioeconomic status, exclusive breast feeding in the first six months *Cryptosporidium,* atypical EPEC, typical EPEC, ETEC, norovirus, adenovirus and *E bieneusi.* **d,** Association between *Giardia* and LAZ at 24 months across all MAL-ED sites in stratified by EED score: low mean EED score (below 33.3rd percentile, *N*=1469), medium mean EED score (between 33.3rd and 66.6th percentile, *N*=1469) and high mean EED score (above 66.6th percentile, *N*=1469). Shown as median difference and 95% confidence interval. Estimates presented are adjusted for enrolment LAZ, sex, socioeconomic status, maternal height, exclusive breastfeeding duration in the first six months of life (left panel) and additionally adjusted for *Campylobacter, Shigella, enteroaggregative Escherichia coli and Enterocytozoon bieneusi* (right panel). All data represent biologically independent samples. Source data are provided as a Source Data file.

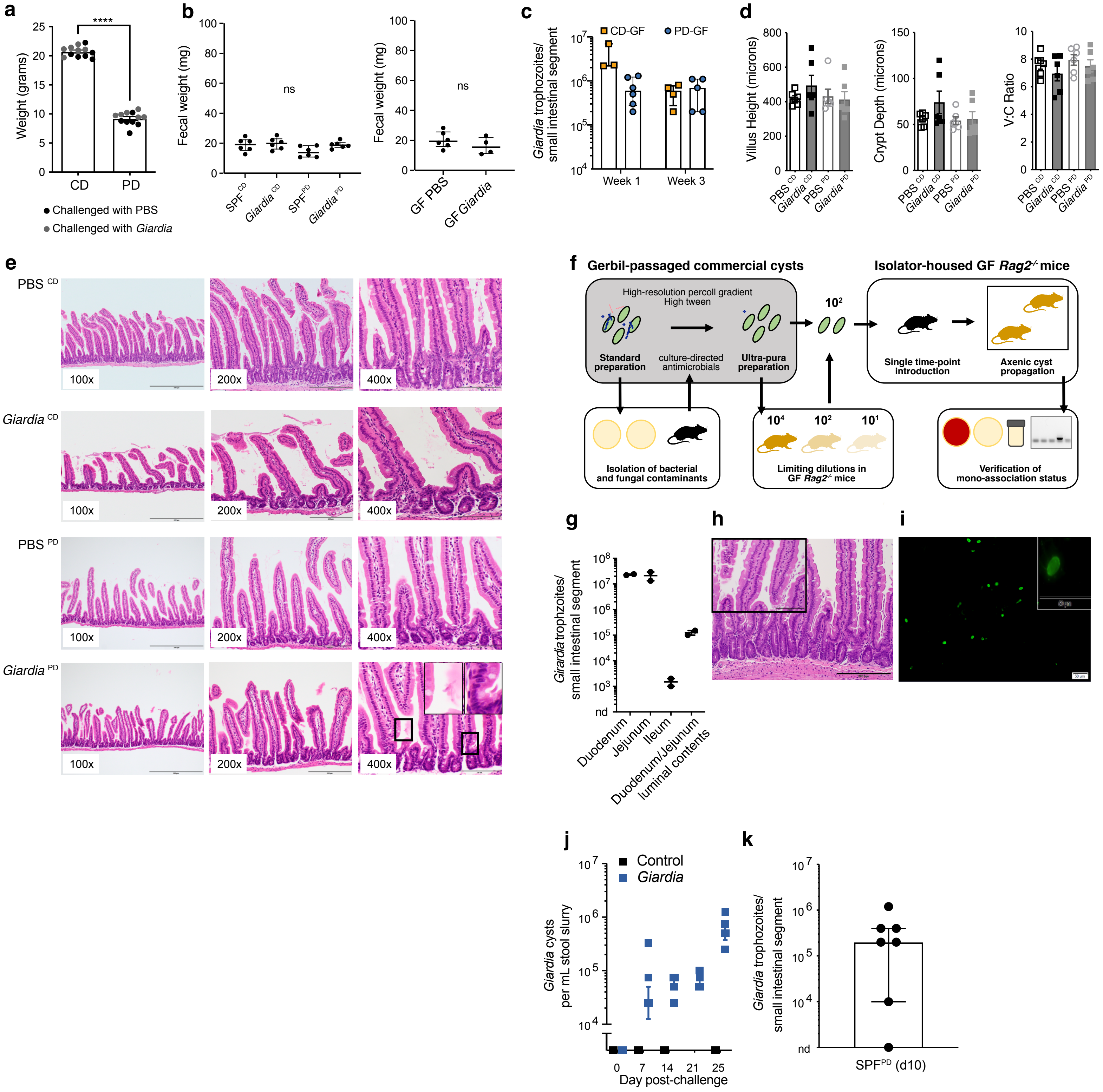
a
Weight (grams)
****
CD
PD
● Challenged with PBS
● Challenged with Giardia
b
Fecal weight (mg)
ns
SPF CD
Giardia CD
SPF PD
Giardia PD
Fecal weight (mg)
ns
GF PBS
GF Giardia
c
Giardia trophozoites/ small intestinal segment
CD-GF
PD-GF
Week 1
Week 3
d
Villus Height (microns)
Crypt Depth (microns)
V:C Ratio
PBS CD
Giardia CD
PBS PD
Giardia PD
e
PBS CD
Giardia CD
PBS PD
Giardia PD
100x
200x
400x
f
Gerbil-passaged commercial cysts
Isolator-housed GF Rag2-/- mice
High-resolution percoll gradient
High tween
Standard preparation
culture-directed antimicrobials
Ultra-pura preparation
10²
Single time-point introduction
Axenic cyst propagation
Isolation of bacterial and fungal contaminants
10⁴
10²
10¹
Limiting dilutions in GF Rag2-/- mice
Verification of mono-association status
g
Giardia trophozoites/ small intestinal segment
nd
Duodenum
Jejunum
Ileum
Duodenum/Jejunum luminal contents
h
i
j
Control
Giardia
Giardia cysts per mL stool slurry
Day post-challenge
k
Giardia trophozoites/ small intestinal segment
nd
SPF PD (d10)

Supplementary Figure 3. Extended observations and validations from experimental *Giardia* challenge outcomes in specific pathogen free and germ-free mice

a, Absolute weights (grams) of C57Bl/6 male mice on the day of *Giardia* challenge (grey dots) or PBS control (black dots) after 10 days on control diet (CD) or protein deficient diet (PD) as in **Figure 2g**. (mean ± SEM, ****P<0.0001, two-sided Unpaired test for comparison between mice on CD (N=12) versus mice on PD (N=12) diet). **b,** Fecal weights in SPF mice (N=6 per group) (left) and GF mice (PBS (N=6) and *Giardia* (N=4)) (right) at the conclusion of each experiment (day 10 after *Giardia* challenge in SPF and day 11 after *Giardia* challenge in GF mice) in different experimental groups as indicated (median ± IQR, Kruskal-Wallis with Dunn's multiple comparisons test). **c,** *Giardia* trophozoites per 4 cm duodenal segment in GF mice on either diet at 1 (CD, N=3; PD, N=5) and 3 weeks (CD, N=4; PD, N=5) after challenge with 10^4 *G. lamblia* (H3) cysts (median ± IQR). **d,** Duodenal villus and crypt morphometry (median ± IQR, Shown are biologically independent samples plotted as the mean taken from at least 10 replicate measured villus-crypt units per mouse, N=6 individual mice per group) and **e,** representative histology from duodenal sections of one representative mouse in each group (CD=control diet, PD=protein deficient diet) on day 11 after *Giardia* or PBS challenge (100x (500 µm scale bar), 200x (200 µm scale bar) and 400x (100 µm scale bar) magnification as indicated). **f,** Schematic for generation of *in vivo* axenic *Giardia* cysts derived from gerbil-passaged ultra-pure commercial *G. lamblia* H3 cyst preparation. **g**, Validation of *Giardia* colonization density (median, N=2 independent mice), **h,** spatial relationship with intestinal mucosa, and **i,** life-cycle stages in the murine intestine. Photomicrographs of duodenum are H/E-stained, 200 x with 200 µm scalebar, inset is 400 x with 100 µm scalebar. Immunofluorescence stain (*Giardia*-a-glo®) *Giardia* cysts: visualized in a mono-

associated mouse, 200x with 50 µm scale bar, inset is 400x with 50 µm scalebar. **j,** cysts per single fecal collection for individual GF mice beginning 7 days after challenge with 10^3 H3 cysts (median ± IQR, N=4 biologically independent mice at each timepoint). **k,** day 10 after challenge with 10^4 axenic *G. lamblia* cysts in 3-week-old PD-diet fed SPF mice (mice were on PD diet for 10 days prior to *Giardia* challenge) (median ± IQR, N=6 biologically independent mice). All data represent biologically independent samples. Source data are provided as a Source Data file.

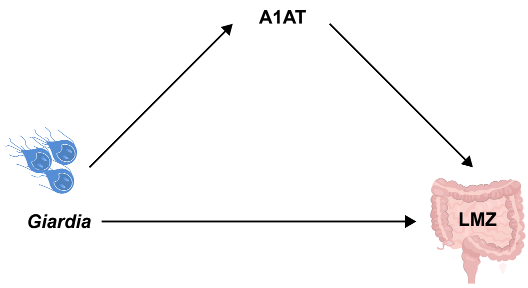

		Estimate	P-value
BGD	ACME	-0.006 (-0.02, 0.01)	0.43
	ADE	0.068 (-0.10, 0.23)	0.37
PEL	ACME	-0.006 (-0.02, 0.00)	0.134
	ADE	0.109 (0.03, 0.19)	**0.002**

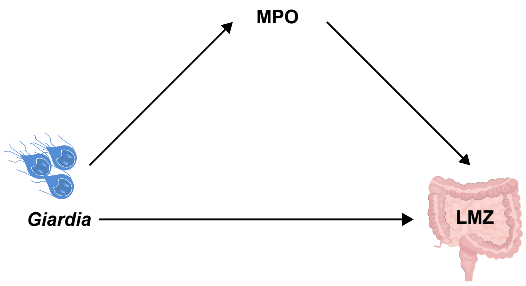

		Estimate	P-value
BGD	ACME	0.000 (-0.02, 0.02)	0.96
	ADE	0.068 (-0.10, 0.23)	0.40
PEL	ACME	-0.001 (-0.07, 0.00)	0.610
	ADE	0.098 (0.02, 0.18)	**0.008**

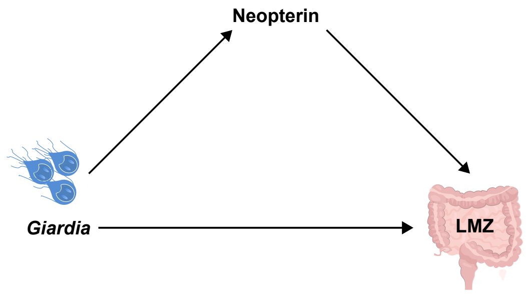

		Estimate	P-value
BGD	ACME	0.002 (-0.01, 0.01)	0.95
	ADE	0.064 (-0.10, 0.23)	0.42
PEL	ACME	-0.001 (-0.07, 0.01)	0.684
	ADE	0.099 (0.02, 0.18)	**0.008**

Supplementary Figure 4: Mediation analyses of *Giardia* and LMZ by levels of fecal EED biomarkers

Mediation analysis revealed that intestinal permeability (expressed as LMZ) as a result of *Giardia* infection in not significantly mediated by the levels of A1AT, MPO and neopterin which are markers of intestinal inflammation and barrier disruption. Covariates included in the modelling process included sex, age and socioeconomic status. Only children with matching A1AT, MPO, neopterin, LMZ and *Giardia* detection data were included in the analysis. To facilitate comparisons between Peru and Bangladesh only matching data from measurements at 3, 6, 9 and 15 months were included in the models since no LMZ measurements were available for Bangladesh at 24 months. All data represents biologically independent samples. Source data are provided as a Source Data file.

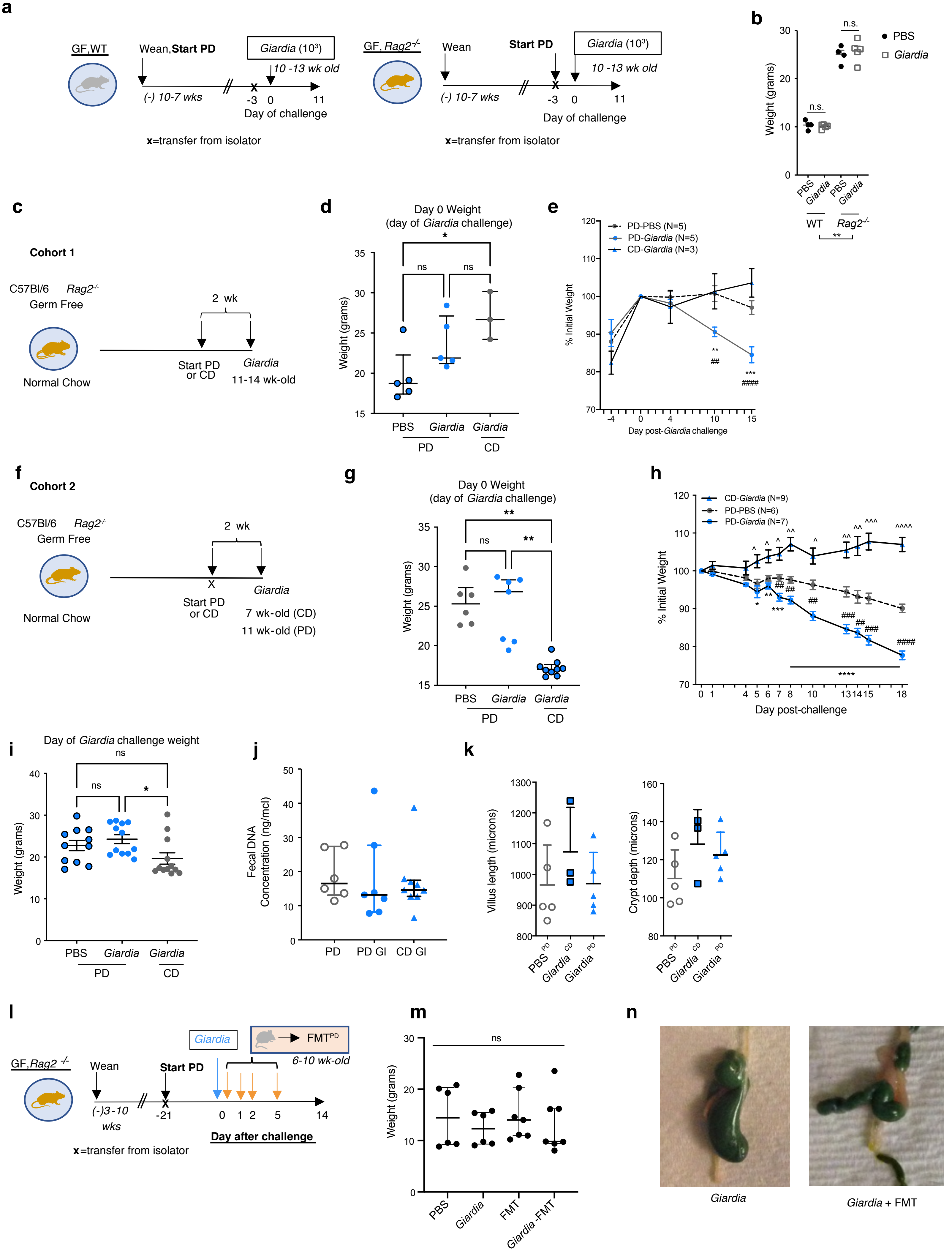

a
GF,WT
Wean, Start PD
Giardia (10³)
10 -13 wk old
(-) 10-7 wks
-3 0 11
Day of challenge
x=transfer from isolator
GF, Rag2-/-
Wean
Start PD
Giardia (10³)
10 -13 wk old
(-) 10-7 wks
-3 0 11
Day of challenge
x=transfer from isolator
b
Weight (grams)
n.s.
n.s.
PBS
Giardia
WT
Rag2-/-
**
c
Cohort 1
C57Bl/6 Rag2-/-
Germ Free
Normal Chow
2 wk
Start PD or CD
Giardia
11-14 wk-old
d
Day 0 Weight
(day of Giardia challenge)
Weight (grams)
*
ns
ns
PBS
Giardia
Giardia
PD
CD
e
PD-PBS (N=5)
PD-Giardia (N=5)
CD-Giardia (N=3)
% Initial Weight
**
##
***
####
Day post-Giardia challenge
f
Cohort 2
C57Bl/6 Rag2-/-
Germ Free
Normal Chow
2 wk
Start PD or CD
Giardia
7 wk-old (CD)
11 wk-old (PD)
g
Day 0 Weight
(day of Giardia challenge)
Weight (grams)
**
ns
**
PBS
Giardia
Giardia
PD
CD
h
CD-Giardia (N=9)
PD-PBS (N=6)
PD-Giardia (N=7)
% Initial Weight
****
Day post-challenge
i
Day of Giardia challenge weight
ns
ns
*
Weight (grams)
PBS
Giardia
Giardia
PD
CD
j
Fecal DNA
Concentration (ng/mcl)
PD
PD GI
CD GI
k
Villus length (microns)
PBS PD
Giardia CD
Giardia PD
Crypt depth (microns)
PBS PD
Giardia CD
Giardia PD
l
GF, Rag2 -/-
Wean
Start PD
Giardia
FMT^PD
6-10 wk-old
(-)3-10 wks
-21
0 1 2 5 14
Day after challenge
x=transfer from isolator
m
ns
Weight (grams)
PBS
Giardia
FMT
Giardia-FMT
n
Giardia
Giardia + FMT

Supplementary Figure 5: Additional experimental details and observations in wild-type and *Rag2*$^{-/-}$ experimental *Giardia* challenge models

a, Experimental timeline for 10-13 week-old male and female germ-free (GF) WT mice (left) and GF *Rag2*$^{-/-}$ mice (right) for mice in **Figure 3d,e. b**, Day-of-challenge absolute weights of GF WT and *Rag2*$^{-/-}$ mice after acclimation to the PD diet for 7-10 weeks (WT) or 3 days (*Rag2*$^{-/-}$) and after 3 days of acclimation to the cubicle-environment **Figure 3d** (mean ± SEM, **P<0.0001, two-sided Unpaired T-test for aggregate WT (N=9; N=4 PBS and N=5 *Giardia*) versus *Rag2*$^{-/-}$ (N=9; N=4 PBS and N=5 *Giardia*). ns=not significant for two-sided Unpaired T-test within each WT or *Rag2*$^{-/-}$ group respectively. **c-e,** Cohort 1 in **Figure 4a,b**. Male and female 11-14 week-old GF *Rag2*$^{-/-}$ mice were fed normal chow until 2 weeks prior to commercial *Giardia* cyst challenge. Simultaneous with initiation on either CD or PD diets, mice were transferred to the cubicle environment. **c,** Experimental timeline. **d,** Day-of-challenge absolute weights after acclimation to the CD or PD diet for 2 weeks. (median ± IQR, *P<0.047 for CD-*Giardia* (N=3) vs PD-PBS (N=5), two-sided Mann-Whitney U-test. PD-*Giardia* (N=5). **e,** Weights as percent change relative to day of challenge (Day 0) beginning 4 days prior to and through 15 days post-challenge (mean ± SEM, $^{\#\#}$P=0.0014 and $^{\#\#\#\#}$P<0.0001 for PD-*Giardia* (N=5) vs CD-*Giardia* (N=3); *P=0.03 and ***P=0.006 for PD-*Giardia* (N=5) vs PD-PBS (N=5), Two-way ANOVA repeated measures, Bonferonni multiple comparisons test). **f-h,** Cohort 2 in **Figure 4a,c**. Male and female 7-11 week-old GF *Rag2*$^{-/-}$ mice were fed normal chow until 2 weeks prior to commercial *Giardia* cyst challenge. Simultaneous with initiation on either CD or PD diets, mice were transferred to the cubicle environment. **f,** Experimental timeline. **g,** Day-of-challenge absolute weights of CD (7-week-old) and PD (11-week-old) diet-fed *Rag2*$^{-/-}$ mice after acclimation to the CD or PD diet for 2 weeks. (median ± IQR,

P=0.004,CD-*Giardia* (N=9) vs either PD-PBS (N=6) or PD-*Giardia* (N=7), Kruskal-Wallis, Dunn's multiple comparisons test. **h, Weights as % change relative to day-of-challenge (Day 0) through 11 days post-challenge. (mean ± SEM, *P<0.05, **P<0.01, ***P<0.001, and ****P<0.0001 for PD-*Giardia* (N=7) vs CD-*Giardia* (N=9); $^{\#\#}$P<0.01, $^{\#\#\#}$P<0.001, $^{\#\#\#\#}$P<0.0001 for PD-*Giardia* (N=7) vs PD-PBS (N=6); ^P<0.05, ^^P<0.01, ^^^P<0.001, ^^^^P<0.0001 for PD-PBS (N=6) vs CD-*Giardia* (N=9), Two-way ANOVA, repeated measures, Bonferonni multiple comparisons test. See Source Data sheet for exact P-values). **i,** Day-of-challenge absolute weights, aggregated by diet in cohort 1 and cohort 2 (median ± IQR, PBS-PD N=11 (N=5 cohort 1 and N=6 cohort 2), PD-*Giardia* N=12 (N=5 cohort 1 and N=7 cohort 2), CD-*Giardia* N=12 (N = 3 cohort 1 and N=9 cohort 2; *=0.02 PD-PBS versus CD-*Giardia*, Kruskal-Wallis, Dunn's multiple comparisons test). **j,** DNA concentration after extraction of fecal pellets from day 18 (cohort 2, PD N=6, PD-*Giardia* N=7, CD-*Giardia* N=9; median ± IQR). **k,** Duodenal villus length and crypt depth on day 15 after *Giardia* challenge (cohort 1, PBS-PD N=5, PD-*Giardia* N=6, CD-*Giaria* N=3; mean ± SEM. Shown are biologically independent samples plotted as the mean taken from at least 10 replicate measured villus-crypt units per mouse). **l-n,** Experiment details for **Figure 4d,e.** Male and Female 3-10-week-old GF *Rag2*$^{-/-}$ mice were transferred to the cubicle environment and started on the PD diet for 21 days prior to challenge with 10^5 commercial purified *Giardia* cysts (N=13) or PBS (N=13). 7 mice in each group were also exposed to serial fecal microbiota transfers (FMT) (day 0, 1, 2, and 5). **l,** Experimental timeline. **m,** Day-of-challenge (day 0) absolute weights in each group (median ± IQR, PBS N=6, PBS-FMT N=7, *Giardia* N=6, *Giardia*-FMT N=7, ns, Kruskal-Wallis, Dunn's multiple tests comparator. **n,** Representative ceca of *Giardia* mono-challenged (left) and *Giardia* + FMT (right)

challenged PD diet fed *Rag2*$^{-/-}$ mice. All data represent biologically independent samples and/or animals. Source data are provided as a Source Data file.

a

source	bacteria (MALDI-TOF)	Yeast (WGS)
Standard Lot 1		
Giardia inoculum *(10^6)*	*E. gallinarum, Rhizobium radiobacter, Lactobacillus spp.,* Unidentified GPR, *Stenotrophomonas maltophilia*	*Candida famata*
Giardia inoculum *(10^3)*	NONE	*C. famata, C. spp.*
Standard Lot 2		
Giardia inoculum *(10^3)*	*E. gallinarum/casseliflavus*<br>*Lactobacillus spp.*<br>*Macrococcus*	*C. sake*
Standard Lot 3		
Giardia inoculum *(10^3)*	*S. maltophilia*	*C. parapsilosis*<br>*C. famata*<br>*Unidentified yeast*
**Unprocessed gerbil stool**	*Enterococcus gallinarum, Staphylococcus aureus,*<br>*S. sylosus, Bacillus spp.*<br>*Pseudomonas fluorescens,*<br>*P. veronii*	NONE
Ultra-Pure Lot 1		
Giardia inoculum (10^4)	consistent with *Enterococcus spp.* (1 and 4 colonies)*	Consistent with *Candida spp.* (51 and 40 colonies)*
Ultra-Pure Lot 2		
Giardia inoculum (10^2)	NONE	

*identification based on colony morphology compared with previous

b

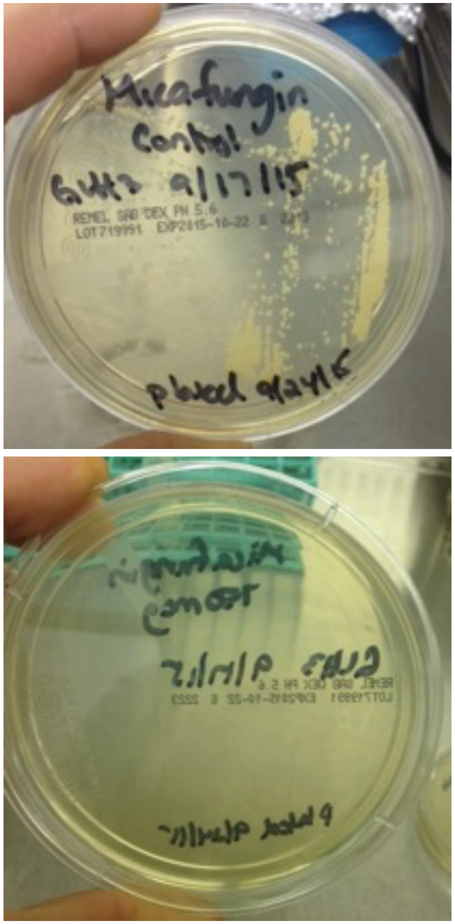

c

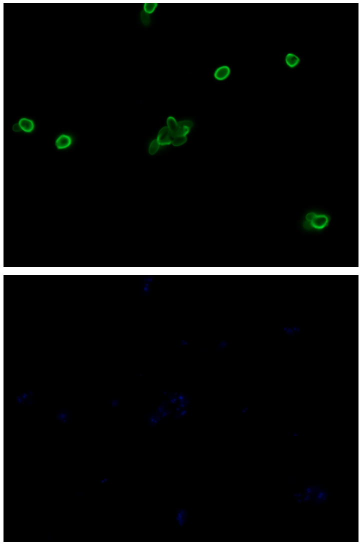

d

Microbe detection, N (%)	Standard prep *Isolator* 10³	Ultra-pure prep *Cubicle* — *Experiment 1* 10³	*Experiment 1* PBS	*Experiment 2* 10⁴	*Experiment 2* 10²	*Experiment 2* 10¹	*Experiment 2* PBS
***Giardia* positive**	4 (100)	10 (100)	0 (0)	8 (100)	5 (63)	0 (0)	0 (0)
**BHI culture pos.**	4* (100)	4** (40)	4*** (50)	1^ (10)	0 (0)	0 (0)	0 (0)
**Sab Dex culture pos.**	0 (0)	0 (0)	0 (0)	0 (0)	0 (0)	0 (0)	0 (0)

**E. gallinarum/casseliflavus*, unidentified gram-positive rod

***Bacillus iriensis, Brevibacillus choshinensis*

****B. iriensis, B.choshinensis, Paenibacillus validus*

^unidentified gram-positive rod

Supplementary Figure 6. Development ultra-pure gerbil-passaged *Giardia* cysts for axenic propagation

a, Detection of culture-viable bacteria and fungi present in 3 different lots of gerbil-passaged purified *Giardia lamblia* (H3) cysts (Waterborne, Inc.). Bacteria were isolated on conventional media (Luria Broth (LB) agar, Brain Heart Infusion (BHI) agar, and Sheep's Blood Agar) at 37°C and fungi were isolated on Sabouraud dextrose agar (SDA) at 24-30°C. Unique colony morphotypes were identified by MALDI-TOF (bacteria) or Whole genome sequencing (WGS) in the UNC-CH Clinical Microbiology Laboratory. **b,** Representative SDA agar plates showing growth of yeast at room temperature 48 hours after inoculation with 10^3 purified *Giardia* cysts in standard transport solutions (control, top) or transport solution supplemented with 1000 µl/mL micafungin (bottom, no growth). **c,** Immunofluorescent staining of *Giardia* cyst wall protein (*Giardia*-a-glo®, Waterborne, Inc.) of ultra-pure cysts (*top*) and ultra-pure cysts after 30 minutes in 10% bleach (*bottom*). 200x, Scale bar = 50 µm. Data is representative of 6 biologically independent samples. **d,** Detection of *Giardia* and/or bacteria (by BHI agar) or yeast (by SDA agar) 7-11 days after challenge of GF mice with 10^3 commercial gerbil-passaged *G. lamblia* (H3) cysts or 10^1 -10^4 Ultra-purified cysts. All data represents biologically independent samples. Source data are provided as a Source Data file. Source data are provided as a Source Data file.

a

Sterility

Fecal
SBA, Thioglycolate, Gram's Stain: 0/38 (samples), 0%
SDA: 0/36 (samples), 0%
16S rDNA PCR: 0/12 (samples), 0%
TYI-S-33: 0/6 (samples), 0%

Small intestine
BHI: 0/4 (samples), 0%
SDA: 0/4 (samples), 0%
TYI-S-33: 0/4 (samples), 0%

b

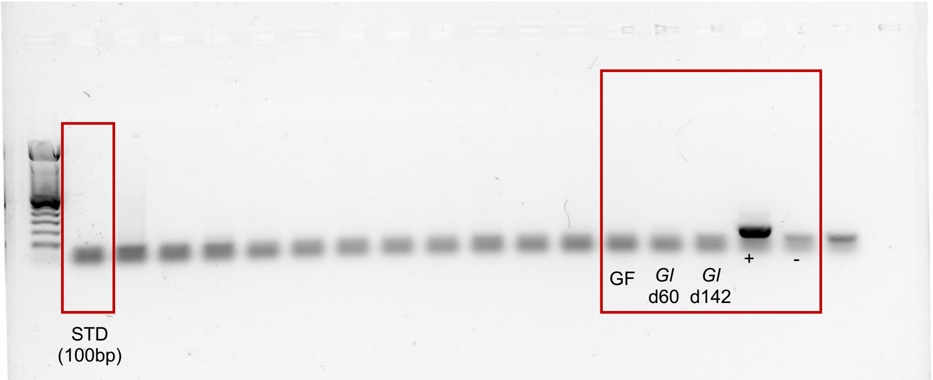

c

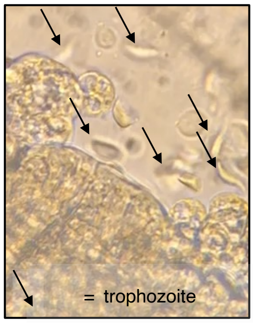

Intestinal mucosa *ex vivo* (40x)

d

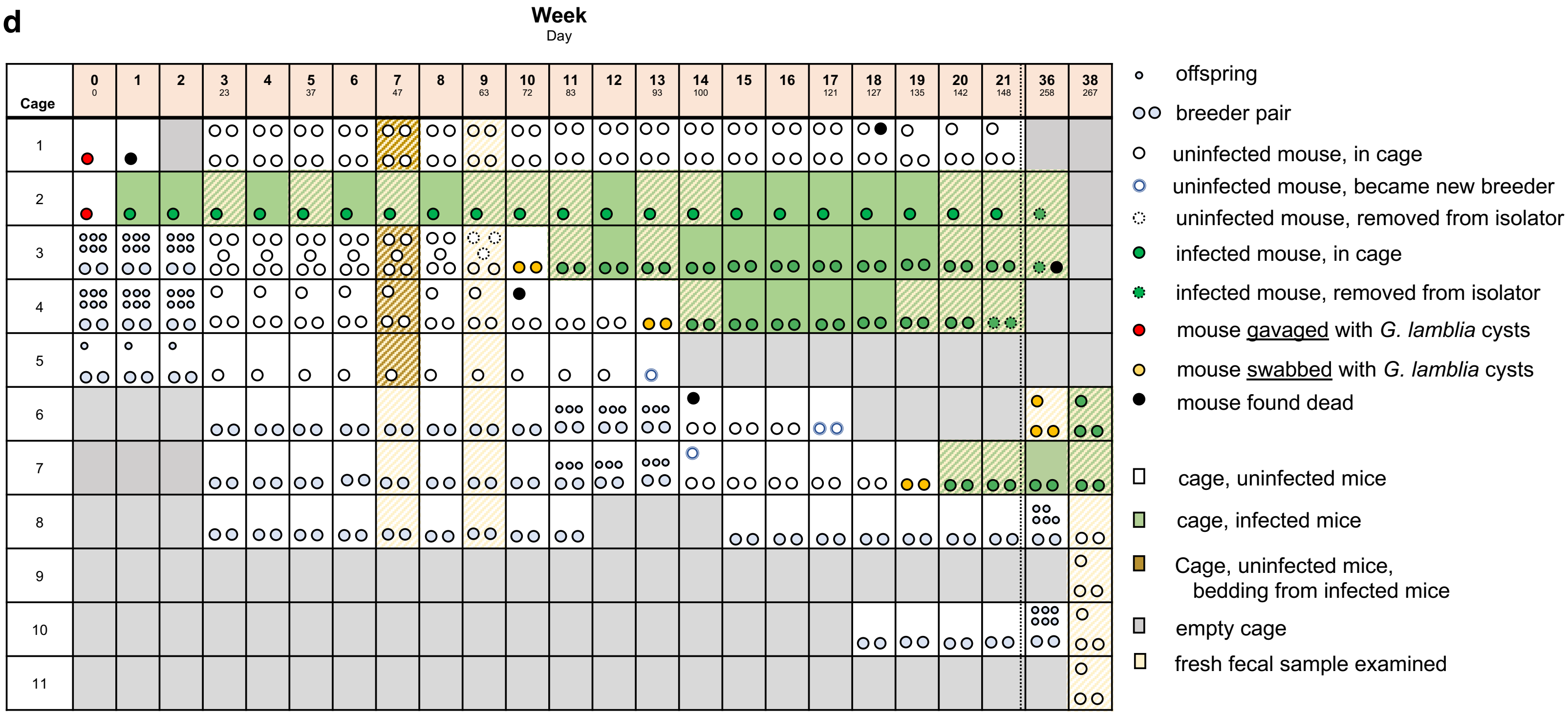

e

Transmissibility

oral gavage (10^2): 1*/2 (mouse), 50%
oral gavage ($\geq 10^3$): 9/9 (mouse), 100%
fecal swab (~10^4/fecal pellet): 9/9 (mouse), 100%
bedding exchange: 0/13 (mouse), 0%
Co-housing (day of infection) 5/6 (mouse), 83%
no direct contact: 0/10 (cage), 0%

Supplementary Figure 7. Validation of *Giardia* mono-association propagation in germ-free isolators

a, Conventional fecal bacteriology (SBA and Thioglycolate), Gram's stain, and 16S rDNA PCR for detecting culture-viable and non-culturable bacteria, SDA for detecting yeast, and TYI-S-33 media without antibiotics for detecting other protozoa in GF *Rag2*$^{-/-}$ mice directly inoculated with 10^2 ultra-pure *G. lamblia* (H3) cysts and propagators secondarily infected in GF isolators. Small intestinal contents were cultured in BHI, SDA, and TYI-S-33 media without antibiotics. (Denominators represent biological replicates (individual mice) of triplicate technical replicates (culture). All samples were obtained between 3-267 days after *Giardia* challenge as indicated in c. **b,** Universal 16S PCR of fecal samples from GF and *Giardia*-mono-associated mice (*Gl*) at 60 and 142 days after propagation as indicated. Positive control = *E. coli*, Negative control (-) = extraction blank (representative gel from monthly (N=11) surveillance samples). Unlabeled lanes are surveillance fecal samples from germ-free mice from a range of isolators in the National Gnotobiotics Rodent Resource Center. **c,** *Giardia* trophozoites in *ex vivo* upper small intestinal fragments. Data is representative of 20 biologically independent samples. **d,** Husbandry and transmissibility table of cages with GF mice and *Giardia* mono-associated mice housed in the same GF isolators. **e,** Summary of routes of *Giardia* transmissibility from *Giardia* mono-associated propagators to separately caged GF mice housed in the same isolator. All data represents biologically independent samples. Source data are provided as a Source Data file.

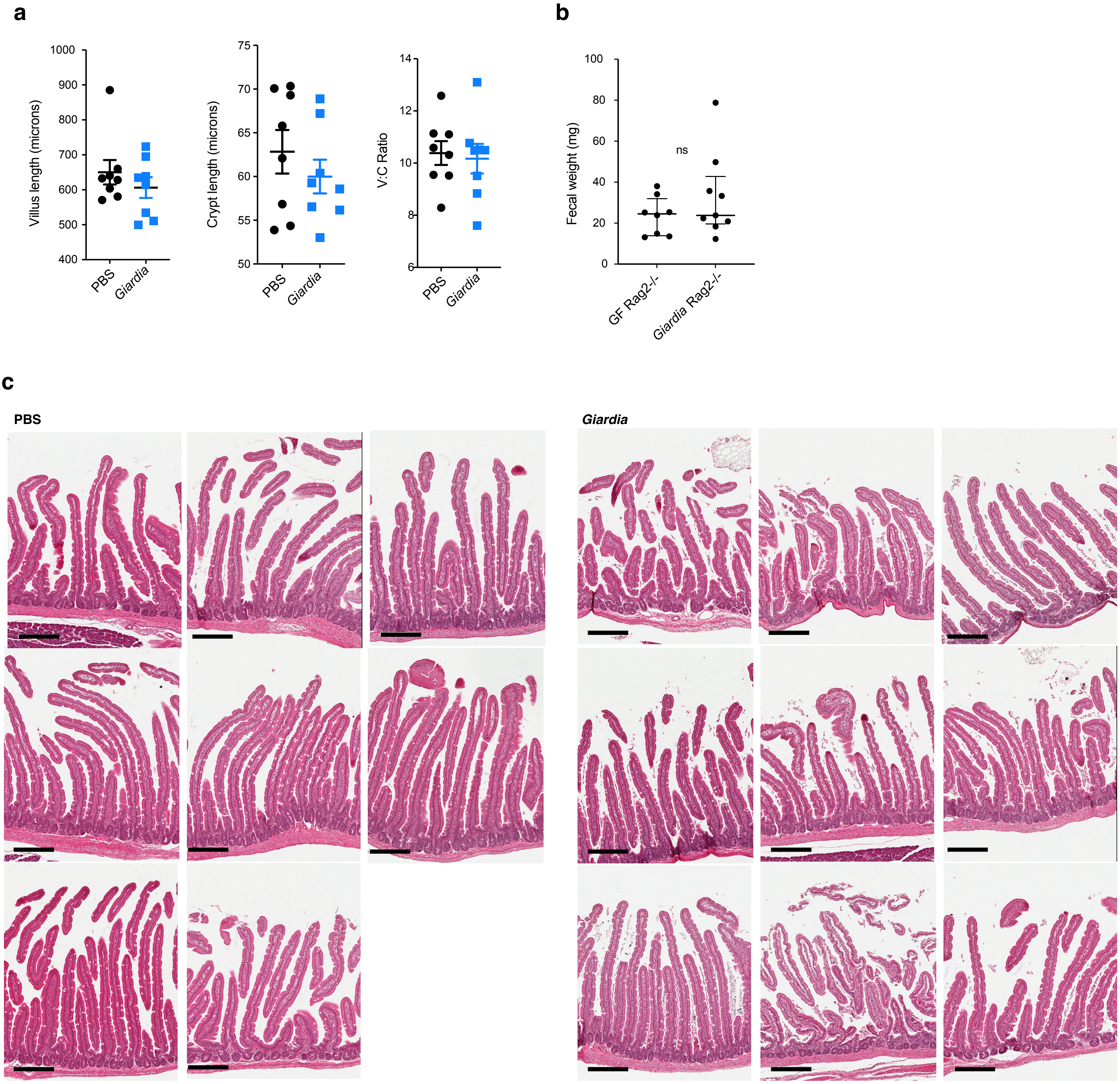

a
b
1000
900
800
700
600
500
400
Villus length (microns)
PBS
Giardia
75
70
65
60
55
50
Crypt length (microns)
14
12
10
8
6
V:C Ratio
100
80
60
40
20
0
Fecal weight (mg)
ns
GF Rag2-/-
Giardia Rag2-/-
c
PBS
Giardia

Supplementary Figure 8. Intestinal morphometry in GF and *Giardia*-mono-associated *Rag2*$^{-/-}$ mice on a protein deficient diet

a, Duodenal villus and crypt morphometry (mean± SEM; PBS N=8 and *Giardia* N=8 (one mouse excluded due to inadequate quality histology)). Shown are biologically independent samples plotted as the mean taken from at least 10 replicate measured villus-crypt units per mouse) and **b,** fecal weights (median ± IQR, PBS N=8 and *Giardia* N=9 biologically independent mice, two-sided Mann-Whitney U-test, non-significant), and **c,** representative histology from duodenal sections of mice in each group (PBS or *Giardia*) as indicated from Figure 3n on day 25 after *Giardia* challenge, 100x, 200 μm scale bar. All data represent biologically independent samples. Source data are provided as a Source Datafile.

a

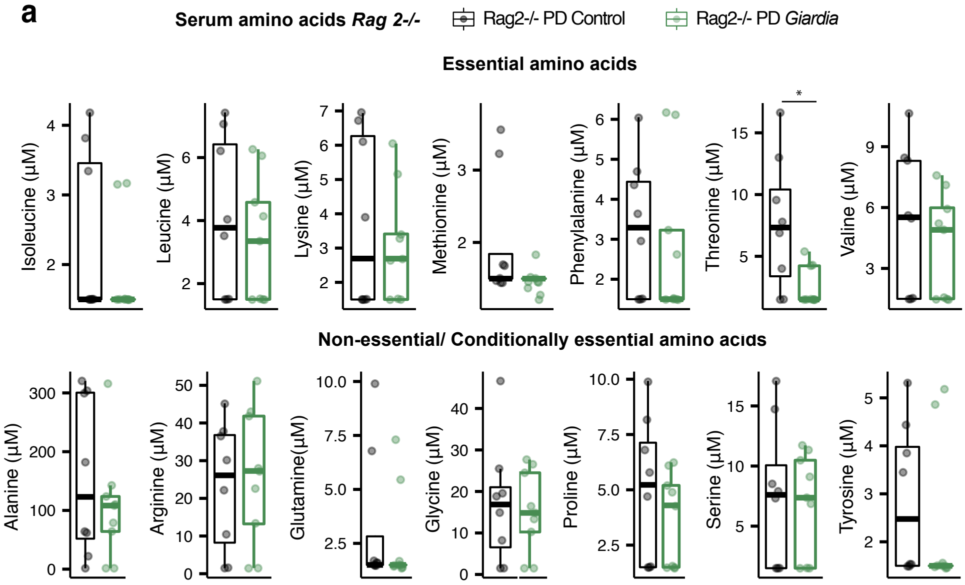

Serum amino acids Rag 2-/-
Rag2-/- PD Control
Rag2-/- PD Giardia
Essential amino acids
Isoleucine (µM)
Leucine (µM)
Lysine (µM)
Methionine (µM)
Phenylalanine (µM)
Threonine (µM)
Valine (µM)
*
Non-essential/ Conditionally essential amino acids
Alanine (µM)
Arginine (µM)
Glutamine(µM)
Glycine (µM)
Proline (µM)
Serine (µM)
Tyrosine (µM)

Supplementary Figure 9: Serum free amino acids in *Rag2*$^{-/-}$ mice

Serum free amino acids day 25 after *Giardia* challenge in *Rag2*$^{-/-}$ GF mono-associated and control PD diet-fed mice (median ± IQR, *P=0.037 for threonine, two-sided Mann-Whitney U-test). All data represents biologically independent samples. Source data are provided as a Source Data file.

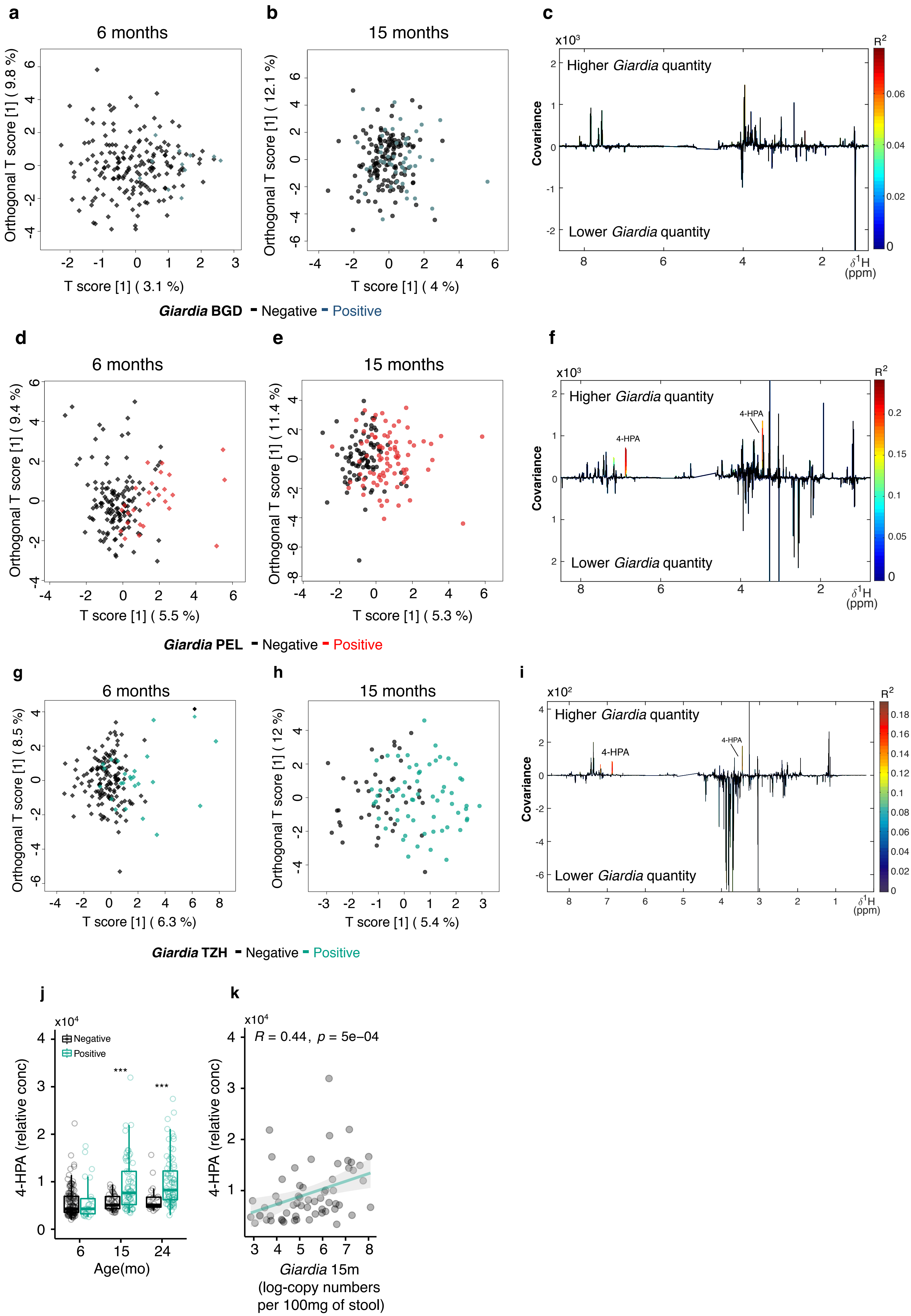

a
6 months
Orthogonal T score [1] (9.8 %)
T score [1] (3.1 %)
b
15 months
Orthogonal T score [1] (12.1 %)
T score [1] (4 %)
c
x10³
Higher Giardia quantity
Lower Giardia quantity
Covariance
R²
δ¹H (ppm)
Giardia BGD - Negative - Positive
d
6 months
Orthogonal T score [1] (9.4 %)
T score [1] (5.5 %)
e
15 months
Orthogonal T score [1] (11.4 %)
T score [1] (5.3 %)
f
x10³
Higher Giardia quantity
4-HPA
4-HPA
Lower Giardia quantity
Covariance
R²
δ¹H (ppm)
Giardia PEL - Negative - Positive
g
6 months
Orthogonal T score [1] (8.5 %)
T score [1] (6.3 %)
h
15 months
Orthogonal T score [1] (12 %)
T score [1] (5.4 %)
i
x10²
Higher Giardia quantity
4-HPA
4-HPA
Lower Giardia quantity
Covariance
R²
δ¹H (ppm)
Giardia TZH - Negative - Positive
j
x10⁴
Negative
Positive
***
***
4-HPA (relative conc)
Age(mo)
k
x10⁴
R = 0.44, p = 5e−04
4-HPA (relative conc)
Giardia 15m
(log-copy numbers
per 100mg of stool)

Supplementary Figure 10. Metabolomics analysis of children in Bangladesh, Peru, and Tanzania

a-c, Orthogonal projections to latent structures – discriminant analysis (OPLS-DA) scores plot comparing urinary metabolic profiles of Bangladeshi children with *Giardia* negative (black) and *Giardia* positive (teal) stools at **a,** 6 months and **b,** 15 months of age. **c,** OPLS regression model derived correlation coefficients plot, identifying metabolic associations with *Giardia* burden in 15-month-old children from Bangladesh. **d-f,** OPLS-DA scores plot comparing urinary metabolic profiles of Peruvian children with *Giardia* negative (black) and *Giardia* positive (red) stools at **d,** 6 months and **e,** 15 months of age. **f,** OPLS regression model derived correlation coefficients plot, identifying metabolic associations with *Giardia* burden in 15-month-old children from Peru. **g-i,** OPLS regression model derived correlation coefficients plot, identifying metabolic associations with *Giardia* burden in 15-month-old children from Tanzania. **g,** OPLS-DA scores plot comparing urinary metabolic profiles of Tanzania children with *Giardia* negative (black) and *Giardia* positive (green) stools at **g,** 6 months and **h,** 15 months of age. **i,** OPLS regression model derived correlation coefficients plot, identifying metabolic associations with *Giardia* burden in 15-month-old children from Tanzania. For all OPLS regression figures, positive peaks indicate metabolites that were excreted in higher amounts in children infected with higher *Giardia* burden and negative peaks indicate metabolites that were excreted in lower amounts. The colour scale represents the degree of correlation of each metabolite to infection status, with red indicating stronger association and blue indicating weaker association whereas black-coloured peaks indicate metabolites not significantly associated with infection status (Benjamini-Hochberg procedure for false discovery rate correction ($p<0.05$)). (**c,f,i**, Model diagnostics: BGD, $R^2X=0.79$, $R2\hat{Y}=0.04$, $Q2\hat{Y}=-0.02$, $P=0.2$, PEL, $R^2X=0.22$, $R2\hat{Y}=0.24$, $Q2\hat{Y}=0.03$, $P=0.004$, TZH: $R^2X=0.46$,

R2Ŷ=0.21, Q2Ŷ=0.05, P=0.08. R2X: fraction of variation of the descriptive matrix explained by the model, R2Ŷ: goodness of fit, Q2Ŷ: predictive ability of the model, P: p-value calculated following permutation testing (1000 permutations)). **j,** Relative concentration of urinary 4-hydryxyphenylacetate (4-HPA) in Tanzanian children (at 6 (N=164), 15 (N=106), and 24 (N=95) months old) with positive or negative *Giardia* detections (median ± IQR, ***P=3.5e-05 at 15 months and ***P=0.00011 at 24 months old two-sided Mann-Whitney U-test comparing children by *Giardia* status). **k**, Correlation between urinary 4-HPA and concurrent *Giardia* at 15 months of age in Tanzania (N=61). Shown are trendlines with 95% confidence interval bands. Two-tailed Spearman's rank correlation was used to calculate correlation coefficients (R) and P-values (p). All data represents biologically independent samples. Source data are provided as a Source Data file.

Supplementary Table 1. Quantitative burdens of eight other stool pathogens at 15 months of age in Bangaldesh and Peru. Values indicated median (IQR). Samples with Ct values <LOD were excluded from this analysis. Two-sided Mann-Whitney U-test was used for all analyses.

	BDG	PEL	P-value
**EAEC**	5.83(4.76-7.21)	6.20(4.90-7.65)	0.2435
Shigella	5.39(4.97-6.40)	5.52(4.79-6.68)	0.9282
Campylobacter	4.98(4.30-5.72)	5.11(4.24-6.73)	0.3548
**Atyp EPEC**	5.11(4.22-6.46)	5.78(4.55-7.18)	0.0843
**Typ EPEC**	6.04(4.98-6.62)	6.02(4.77-7.13)	0.9234
**LT-ETEC**	4.47(4.02-6.11)	4.24(3.92-6.59)	0.9128
**ST-ETEC**	4.74(3.73-6.67)	4.53(4.13-6.79)	0.9875
Cryptosporidium	5.18(4.13-6.11)	5.25(4.43-6.12)	0.8731
