# Supplementary material for: Giardia hinders growth by disrupting nutrient metabolism independent of inflammatory enteropathy
Source: Nat Commun. 2023 May 18;14:2840. doi: 10.1038/s41467-023-38363-2 (PMC10195804; doi:10.1038/s41467-023-38363-2)
Supplement: Supplementary file 2 — Reporting Summary [file 41467_2023_38363_MOESM2_ESM.pdf]

nature portfolio

Corresponding author(s): Luther A. Bartelt
Natasa Giallourou

Last updated by author(s): 3/30/2023

Reporting Summary

Nature Portfolio wishes to improve the reproducibility of the work that we publish. This form provides structure for consistency and transparency in reporting. For further information on Nature Portfolio policies, see our Editorial Policies and the Editorial Policy Checklist.

Statistics

For all statistical analyses, confirm that the following items are present in the figure legend, table legend, main text, or Methods section.

n/a	Confirmed	
☐	☒	The exact sample size (*n*) for each experimental group/condition, given as a discrete number and unit of measurement
☐	☒	A statement on whether measurements were taken from distinct samples or whether the same sample was measured repeatedly
☐	☒	The statistical test(s) used AND whether they are one- or two-sided<br>*Only common tests should be described solely by name; describe more complex techniques in the Methods section.*
☐	☒	A description of all covariates tested
☐	☒	A description of any assumptions or corrections, such as tests of normality and adjustment for multiple comparisons
☐	☒	A full description of the statistical parameters including central tendency (e.g. means) or other basic estimates (e.g. regression coefficient) AND variation (e.g. standard deviation) or associated estimates of uncertainty (e.g. confidence intervals)
☐	☒	For null hypothesis testing, the test statistic (e.g. *F*, *t*, *r*) with confidence intervals, effect sizes, degrees of freedom and *P* value noted<br>*Give P values as exact values whenever suitable.*
☒	☐	For Bayesian analysis, information on the choice of priors and Markov chain Monte Carlo settings
☒	☐	For hierarchical and complex designs, identification of the appropriate level for tests and full reporting of outcomes
☐	☒	Estimates of effect sizes (e.g. Cohen's *d*, Pearson's *r*), indicating how they were calculated

Our web collection on statistics for biologists contains articles on many of the points above.

Software and code

Policy information about availability of computer code

Data collection	De-identified data from the MAL-ED cohort were collated in Microsoft Excel and Microsoft Access and mouse experiment data was recorded and collated in Microsoft Excel.
Data analysis	Human data were analyzed using SAS version 9.4 and using the PROC GENMOD package, R version 4.0.2 with RStudio version 1.2.1335 and using the R mediation analysis package. NMR metabolomics data were processed using the Imperial Metabolic Profiling and Chemometrics Toolbox (https://github.com/csmsoftware/IMPaCTS) on Matlab version 2018a. Data from the animal studies were analyzed on GraphPad Prism version 9.3.

For manuscripts utilizing custom algorithms or software that are central to the research but not yet described in published literature, software must be made available to editors and reviewers. We strongly encourage code deposition in a community repository (e.g. GitHub). See the Nature Portfolio guidelines for submitting code & software for further information.

Data

Policy information about availability of data

All manuscripts must include a data availability statement. This statement should provide the following information, where applicable:

- Accession codes, unique identifiers, or web links for publicly available datasets
- A description of any restrictions on data availability
- For clinical datasets or third party data, please ensure that the statement adheres to our policy

All source data for the results reported in this study are provided in the Source Data file. The metabolomics data generated in this study were identified using the Human Metabolome Database and have been deposited on Zenodo under the access link ZENODO LINK. Additional individually-linked data underlying the results presented in the study are available from the ClinEpiDB database (https://clinepidb.org/ce/app/workspace/analyses/DS_5c41b87221/new/details). One must register and request access to download data; data can be downloaded after a committee reviews the request and grants access. All disaggregated individually-linked raw data for all mouse experimentation are present in the Source Data file.

Human research participants

Policy information about studies involving human research participants and Sex and Gender in Research.

Reporting on sex and gender	The study included both males and females. All relevant statistical models were adjusted for sex
Population characteristics	Population included neonates recruited from birth and followed up to 24 months of age. The study included both males and females
Recruitment	The present manuscript reports only analyses from de-identified data in the MAL-ED cohort, and therefore no participants were actively recruited for the present manuscript. Briefly, in the parent MAL-ED study, each site aimed to recruit at least 200 children over a 24-month time period - targeting a smooth and constant monthly enrollment across this time frame - to assess seasonal effects of exposures and to minimize seasonal recruitment biases. Local study personnel met with community leaders and members and recruitment was targeted to pregnant women or very recent mothers (within 20 days of delivery) with careful attention to exclusion criteria to limit perinatal complication biases (such as severe disease requiring hospitalization, severe acute or chronic conditions in maternal-infant dyads diagnosed by a physician, congenital disorders, etc.). Strategies to minimize selection biases included shared protocols with defined inclusion/exclusion criteria, identification of site-specific defined catchment areas, recruitment of all eligible pregnant women (at small density population sites), or a randomization process using Microsoft Excel (at larger density populations sites). For potential bias due to attrition, sites attempt to enroll > 200 participants each. For eligible mother-infant dyads, informed consent was obtained prior to enrollment and data gathering.
Ethics oversight	For the parent MAL-ED study For the parent study, ethical approval was obtained from the institutional review boards at the University of Virginia School of Medicine (Charlottesville, VA) (14595), and at each of the participating research sites: Ethical Review Committee, International Centre for Diarrhoeal Disease Research, Bangladesh (Bangladesh); Committee for Ethics in Research, Universidade Federal do Ceara, and National Ethical Research Committee, Health Ministry, Council of National Health (Brazil); Institutional Review Board, Christian Medical College, Vellore, and Health Ministry Screening Committee, Indian Council of Medical Research (India); Institutional Review Board, Institute of Medicine, Tribhuvan University, Ethical Review Board, Nepal Health Research Council, and Institutional Review Board, Walter Reed Army Institute of Research (Nepal); Institutional Review Board, Johns Hopkins University, and PRISMA Ethics Committee; Health Ministry, Loreto (Peru); Ethical Review Committee, Aga Khan University (Pakistan); Health, Safety and Research Ethics Committee, University of Venda, and Department of Health and Social Development, Limpopo Provincial Government (South Africa); and Medical Research Coordinating Committee, National Institute for Medical Research, and Chief Medical Officer, Ministry of Health and Social Welfare (Tanzania). The study was done in accordance with the principles stated in the Declaration of Helsinki and Good Clinical Practice guidelines. Signed informed consent was obtained by child guardians for each child participant. No monetary compensation was provided for participation. Deidentified participant data from the MAL-ED study is publicly available at ClinEpiDB.org after approval of a proposal by the study's principal investigators (PIs).<br><br>In addition, a Research Consortium Agreement (RCA) was developed and adopted by all collaborating investigators and their institutions in the MAL-ED Consortium prior to the onset of data collection and/or sharing. The RCA provided the organizational framework for the project including management and authorities, governance structure, methods of dispute resolution, and authority of the Network and its associated advisory committees. The RCA also provided guidance on publication; intellectual property; and data ownership, sharing, and release policies. The intent of these policies is to ensure that the important findings resulting from the study are used to benefit those in low-income countries who are most affected. Clearly delineating these issues, with input from the participating institutions and investigators prior to study initiation, was important to effectively establishing harmonization of the study; having the document in place has helped to facilitate the addition of other studies as companion projects. All sites received ethical approval, as appropriate, from governmental, local institutional, and collaborating institutional ethical review boards.

Note that full information on the approval of the study protocol must also be provided in the manuscript.

Field-specific reporting

Please select the one below that is the best fit for your research. If you are not sure, read the appropriate sections before making your selection.

☒ Life sciences ☐ Behavioural & social sciences ☐ Ecological, evolutionary & environmental sciences

For a reference copy of the document with all sections, see nature.com/documents/nr-reporting-summary-flat.pdf

Life sciences study design

All studies must disclose on these points even when the disclosure is negative.

Sample size	Human studies: The present study used all data accessible through the MAL-ED archived de-identified data set. The original MAL-ED protocol targeted enrollment to >200 children per site. These sample sizes were established in the original MAL-ED protocol. To maximize the completeness of our analysis, we therefore used all de-identified data available without any statistical method to predetermine sample size for the analysis in the present study.<br>Mouse studies: For novel gnotobiotic experiments, preliminary data was not available for sample size calculation. Therefore, sample sizes were determined to achieve statistically significant differences for the primary outcome of a 50% growth attainment reduction in Giardia-infected mice 2 weeks after Giardia challenge based on prior experiments (Sample Size calculation for 10 +/- 2.5% mean growth attainment in control mice vs 5% mean growth attainment in Giardia infected mice at beta=80% and alpha <0.05; n=4/group) (see Bartelt, et al. PLoSPathogens, 2017, 13(7): e1006471, Fig. 1 and Fig. 2). Whenever additional age-matched litter-mates were available, but not designated for other purposes, these mice were included in experiments to further improve confidence in estimates of between-group differences and to best utilize the restricted resource of germ-free mice. All efforts were made to limit the total number of mice necessary.
Data exclusions	Human studies: Bias was identified in a subset of Pakistan length measurements; therefore, Pakistan data have been excluded from analyses presented in Extended Data Figure 1.<br>Mouse studies: Due to the small size of protein-deficient diet fed mice, and challenges in handling small mice inside germ-free isolators, there is greater risk of significant trauma at the time of oral gavage. As growth attainment was the most common primary outcome of experiments, we pre-determined that any mice experiencing oral gavage trauma significant enough to cause rapid weight loss with other signs of immediate animal distress (within 24 hours of gavage) would be removed from the experiment and their data excluded. Across all experiments, this occurred in 2 mice and their data was excluded.
Replication	All experimental findings from were verified for reproducibility. If access to germ-free isolators was limited, experiments in germ-free mice were first performed in individually ventilated cages in an SPF barrier facility and then within isolators to confirm findings. When relevant, each of these experiments is reported independently (for example Fig. 2l and Fig. 3d). If repeat experiments were performed in the same environment, then the combined data is shown in the manuscript with each individual experiment detailed in supplemental materials (for example Fig. 3f).
Randomization	Human studies: MAL-ED was an observational study. Recruitment did use a randomizer in high-density population sites. Samples were analysed in a random manner.<br>Mouse studies: For mouse experiments, mice were allocated into dietary and experimental Giardia challenge groups to ensure equal distribution of ages, sex, and weights into each group.
Blinding	Human studies: This is not relevant as MAL-ED was an observational study.<br>Mouse studies: For proper and safe handling purposes to follow stringent gnotobiotic techniques, investigators were not blinding to group allocation during data collection. For data analysis, primary data collectors were blinded to experimental groups (eg. Giardia trophozoite enumerations, histology and microscopy, bioassays) and data as unblinded for statistical comparisons between groups.

Reporting for specific materials, systems and methods

We require information from authors about some types of materials, experimental systems and methods used in many studies. Here, indicate whether each material, system or method listed is relevant to your study. If you are not sure if a list item applies to your research, read the appropriate section before selecting a response.

Materials & experimental systems

n/a	Involved in the study	
☒	☐	Antibodies
☒	☐	Eukaryotic cell lines
☒	☐	Palaeontology and archaeology
☐	☒	Animals and other organisms
☒	☐	Clinical data
☒	☐	Dual use research of concern

Methods

n/a	Involved in the study	
☒	☐	ChIP-seq
☒	☐	Flow cytometry
☒	☐	MRI-based neuroimaging

Animals and other research organisms

Policy information about studies involving animals; ARRIVE guidelines recommended for reporting animal research, and Sex and Gender in Research

Laboratory animals	Mus musculus, C57Bl/6 wild-type and Rag2-/-, male and female, 3-16 weeks of age
Wild animals	No wild animals were used in this study.
Reporting on sex	Study included both make and female mice
Field-collected samples	No field collected samples were used in this study.
Ethics oversight	The animal study protocol was approved by the Institutional Animal Care and Use Committee at the University of North Carolina at Chapel Hill (IACUC protocol #18-266).

Note that full information on the approval of the study protocol must also be provided in the manuscript.
